# Marine probiotics: increasing coral resistance to bleaching through microbiome manipulation

**DOI:** 10.1038/s41396-018-0323-6

**Published:** 2018-12-05

**Authors:** Phillipe M. Rosado, Deborah C. A. Leite, Gustavo A. S. Duarte, Ricardo M. Chaloub, Guillaume Jospin, Ulisses Nunes da Rocha, João P. Saraiva, Francisco Dini-Andreote, Jonathan A. Eisen, David G. Bourne, Raquel S. Peixoto

**Affiliations:** 1 Institute of Microbiology, Federal University of Rio de Janeiro (UFRJ), Rio de Janeiro, Brazil; 2 IMAM-AquaRio – Rio de Janeiro Aquarium Research Center, Rio de Janeiro, Brazil; 3 Instituto de Química, Universidade Federal do Rio de Janeiro (UFRJ), Rio de Janeiro, Brazil; 4 Genome Center, University of California, Davis, CA USA; 5 Department of Environmental Microbiology, Helmholtz Centre for Environmental Research – UFZ, Leipzig, Germany; 6 Department of Microbial Ecology, The Netherlands Institute of Ecology (NIOO-KNAW), Wageningen, The Netherlands; 7 Evolution and Ecology, University of California, Davis, CA USA; 8 Medical Microbiology and Immunology, University of California, Davis, CA USA; 9 College of Science and Engineering, James Cook University, Townsville, Australia; 10 Australian Institute of Marine Science, Townsville, Australia

**Keywords:** Microbial ecology, Climate-change ecology

## Abstract

Although the early coral reef-bleaching warning system (NOAA/USA) is established, there is no feasible treatment that can minimize temperature bleaching and/or disease impacts on corals in the field. Here, we present the first attempts to extrapolate the widespread and well-established use of bacterial consortia to protect or improve health in other organisms (e.g., humans and plants) to corals. Manipulation of the coral-associated microbiome was facilitated through addition of a consortium of native (isolated from *Pocillopora damicornis* and surrounding seawater) putatively beneficial microorganisms for corals (pBMCs), including five *Pseudoalteromonas* sp., a *Halomonas taeanensis* and a *Cobetia marina*-related species strains. The results from a controlled aquarium experiment in two temperature regimes (26 °C and 30 °C) and four treatments (pBMC; pBMC with pathogen challenge – *Vibrio coralliilyticus*, VC; pathogen challenge, VC; and control) revealed the ability of the pBMC consortium to partially mitigate coral bleaching. Significantly reduced coral-bleaching metrics were observed in pBMC-inoculated corals, in contrast to controls without pBMC addition, especially challenged corals, which displayed strong bleaching signs as indicated by significantly lower photopigment contents and *F*_*v*_/*F*_*m*_ ratios. The structure of the coral microbiome community also differed between treatments and specific bioindicators were correlated with corals inoculated with pBMC (e.g., *Cobetia* sp.) or VC (e.g., *Ruegeria* sp.). Our results indicate that the microbiome in corals can be manipulated to lessen the effect of bleaching, thus helping to alleviate pathogen and temperature stresses, with the addition of BMCs representing a promising novel approach for minimizing coral mortality in the face of increasing environmental impacts.

## Introduction

Shallow-water tropical corals build the structural framework that supports the enormous macro- and microbial biological diversity found in reef ecosystems [1, 5]. Despite their key ecological role, coral reefs are threatened by many global impacts, including increasing seawater temperatures that push corals beyond their thermal thresholds, resulting in the breakdown of the close symbiotic interaction between the hosts and their photosynthetic dinoflagellate partners (*Symbiodium*). The ecological disruption of this symbiotic partnership manifests as bleaching, and recent global bleaching events have resulted in extensive loss of reef habitat [3, 6–8]. The Intergovernmental Panel on Climate Change (IPCC) predicts rising global temperatures between 0.4 °C and 1.1 °C by 2025, and, in the most pessimistic scenario, up to 5.8 °C by 2100 (IPCC, 2013), potentially resulting in a catastrophic impact on reef ecosystems [9].

Management priorities for coral reefs have moved beyond documenting their declines, toward investigating potential approaches for mitigating climate impacts, for instance by enhancing coral resistance and resilience using approaches such as assisted recovery and restoration [10–13]. While global action on climate combined with local efforts to actively protect corals from impacts remains a priority, the dire outlook for coral reefs over the coming century has necessitated investigation of other approaches to build resistance and/or resilience into coral populations. Introduction of selectively bred resistant corals has been proposed as a novel approach; however, the native corals’ ability to cope with predicted warming and other environmental stresses (e.g., pathogens) also needs investigation [13].

Microorganisms are key players supporting the functioning and health of multi-cellular life and the ecosystems in which they thrive. Microbiome engineering, i.e., microbial composition and/or function changes, can be achieved through different approaches with these strategies reviewed elsewhere [14]. Manipulation of microbiome communities has been postulated as a key strategy to ‘engineer’ and manipulate host phenotypes and ecosystem functioning [14]. Microbiome engineering has also been indicated as an important strategy for the “smart farming” concept [15], which applies technology to improve agricultural quality, quantity, and sustainability [16]. Such a strategy has been used to improve sustainable agricultural practices [17–19], including the use of biological control of agricultural pests, i.e., the application or manipulation of living biological agents to control plant pathogens and/or insects [20], and the use of Plant Growth-Promoting Rhizobacteria (PGPRs) [21] to improve plant development [22–24]. These are key approaches supporting agricultural activities globally [25], as well as the use of specific probiotics for humans [26]. Bioaugmentation, which consists of increasing the numbers of native bacteria to perform bioremediation of pollutants [27], has been successfully applied to protect corals against oil spills [28]. Although manipulation of microbiomes has been widely (and successfully) used for terrestrial ecosystems (including probiotics for humans [26], agriculture [21–25], and bioremediation [27]), microbiomes are largely underexploited in marine ecosystems.

The manipulation of microbiomes associated with corals (Beneficial Microorganisms for Corals, BMC) [29] was recently proposed as a promising (albeit yet-to-be-explored) tool to improve coral health, potentially promoting resistance and resilience in coral populations and ultimately aiding recovery of impacted reefs [29–32]. BMCs can enhance coral fitness through their symbiotic relationships with the host, including the cycling of nutrients within the holobiont [33–35] or antagonism/exclusion of potential pathogens (biological control) [36–38]. Here, we refer to these organisms as BMCs when their beneficial effects are known. When the effects on coral are simply hypothesized but not yet established, we refer to these organisms as “putative beneficial microorganisms for coral” (pBMCs). BMCs can both form a consistent part of the native coral microbiome and/or be acquired from the surrounding water during adverse environmental conditions [29]. Environmental conditions can shift the coral microbiome by shuffling the relative abundance of existing microbial community members or switching where the microbial communities are exchanged. These changes in microbiome assemblages may also be passed on faithfully through generations to increase host fitness a process termed microbiome-mediated transgenerational acclimatization (MMTA) [39]. The ability to manipulate the coral microbiome through the addition of BMCs, aiming to improve coral resistance to environmental stresses, needs a detailed investigation to establish the effectiveness of this approach, the potential costs, and the exact mechanisms of interaction between the BMCs and the coral host. Here, we describe a successful manipulation of the coral microbiome through the addition of pBMCs that significantly improved coral resistance when exposed to increased seawater temperature, and mitigated the physiological impact on corals challenged with the temperature-dependent pathogen *Vibrio coralliilyticus* (VC).

## Materials and methods

### Ethics approval and consent to participate

The microbial survey permit was obtained from CNPq (Brazilian National Council for Scientific and Technological Development).

### Isolation of BMCs

Three colonies of *Pocillopora damicornis* were used as the source material for isolation of pBMCs. Corals colonies were originally from the Indo-Pacific Ocean. After collection, they were cultured in aquariums from the Coral Vício Company (Peruíbe, Brazil), Robson Aragão Aquariums Nova Friburgo (Rio de Janeiro, Brazil) Leonardo Carvalho, Igor Albergaria, and Marcelo Cunha Aquariums (Rio de Janeiro). We do not have information about the exact geographic location from where these colonies were taken. Samples from all these sources were used for the isolation of pBMC and the experimental set-up. Corals were kept for 60 days in a 390 L aquarium with artificial seawater (Red Sea Salt, Red Sea, USA), and a water flow rate of 10,000 L per hour at the temperature of 26 °C and pH of 8.1. Triplicate macerates obtained from each coral colony (ca. 1 g) and triplicate 100 µL samples of the surrounding water were suspended in 0.85% sterile saline solution (9 mL) and then shaken with glass beads for 16 h. Subsamples (100 µL) of 10^–3^, 10^–4^, and 10^–5^ dilutions were inoculated into Petri dishes containing 20 mL of marine agar (MA) medium (Marine Agar Zobell 2216, Himedia Laboratories, Mumbai, India). A total of 53 bacterial colonies were isolated based on colony morphology, with 30 isolates derived from macerated slurries of *P. damicornis* and 23 isolates from the surrounding water. The collection of isolates was screened for selected pBMC functional attributes [29].

The *Vibrio coralliilyticus* (VC) YB strain (DSM19607), a temperature-dependent pathogen of the coral *P. damicornis* [40, 41], was purchased from DMSZ (Deutsche Sammlung von Mikroorganismen und Zellkulturen GmbH, Germany), and used in subsequent aquarium experiments. This pathogen was also used in antagonistic assays with selected bacterial strains. The strain was stored in 80% glycerol at −80 °C and subsequently recovered and grown on MA medium at 28 °C for 16 h for experimental trials.

### Screening for pBMC traits

The screening for pBMCs was performed based on previously established protocols [29] with selected pBMC traits consistent with the focus of the experiment, i.e., protection against thermal and disease stresses. This included catalase producers to reduce the concentration of reactive oxygen species (ROS), nitrogen cycling, dimethylsulfoniopropionate (DMSP) degradation, and antagonistic bacteria to promote the biological control of pathogens.

Coral-derived bacterial isolates with antagonistic activity against *V. coralliilyticus* were identified through the agar-diffusion method described by Giambiagi-de Marval and colleagues (1990) [42]. Briefly, 20 μL of each bacterial strain was spot-inoculated in a Petri dish containing 3% MA medium. Six ‘spots’ were placed in each Petri dish, each spot representing a different isolate. Plates were incubated at 28 °C for 16 h, after which the plates were inverted on sterile aluminum foil and 1 mL of chloroform-soaked cotton was placed on the inner surface of each dish for 30 min. The antagonistic activity was determined using VC YB as an indicator strain, previously cultured in 3% MB medium (Marine Broth 2216, Himedia Laboratories) and inoculated (1 μL mL^-1^) in 3% MA semi-solid medium, to be poured on the surface of the plates containing the colonies previously inactivated with chloroform. These plates were incubated at 28 °C for 16 h, and the production of antimicrobials was indicated by inhibition halos around the colony spots. Inhibition halos ≥5 mm were considered indicative of inhibitory activity.

All strains were tested for the production of catalase [43]. Briefly, 50 µL of 3% (v/v) hydrogen peroxide was deposited on a microscope slide and mixed with 0.5 mL of the liquid culture of the test microorganism, previously grown at 28 °C for 16 h. If bubbles appeared, the organism was considered catalase-positive, and classified as + (ca. 25% of the surface covered with bubbles), + + (ca. 50% of the surface covered with bubbles), + + + (ca. 75% of the surface covered with bubbles), or + + + + (ca. 100% of the surface covered with bubbles).

### BMC-PCR screening and bacterial 16 S rRNA gene sequencing

Bacteria involved in nutrient cycling, which may improve coral fitness through nitrogen fixation or sulfur cycling (e.g., DMSP degradation), were also selected based on the gene repertoire. Total genomic DNA was isolated from each coral-derived bacterial isolate, using the Wizard Genomic DNA Purification kit (Promega, Madison, WI, USA). Subunits of the nitrogenase (*nifH)*, nitrification (*nirK)* and DMSP degradation *(dmdA)* gene complexes were PCR-amplified from the genomic DNA samples using PCR. For the *nifH* gene, we used the primer set PolF (5′-TGC GAT CCG AAA GCC GAC TC-3′) and PolR (5′-ATG GCC ATC ATT TCA CCG GA-3′) [44]. For the *nirK* gene, we used the primer set F1aCu (5′-ATC ATG GTC CTG CCG CG-3′) and R3Cu (5′-TTG GTG TTA GAC TAG CTC CG-3′) [45]. For the *dmdA* gene, we used the primer set D/all–spFP (5′-TAT TGG TAT AGC TAT-3′) and D/all–spRP (5′-TAA ATA AAA GGT AAA TCG C-3′) [46]. The PCR was performed using 5 µL of 10X buffer, 2.0 mM MgCl_2_, 0.2 mM dNTPs, 5 mM of each primer, ca. 2–4 ng of genomic DNA, and 2.5 U Taq DNA polymerase (Promega), in a final volume of 50 µL. The thermal-cycling protocols were as follows: *nifH*, 94 °C for 3 min; 30 cycles of 94 °C for 1 min, 55 °C for 1 min, and 72 °C for 2 min; and a final extension cycle of 10 min at 72 °C; *nirK*, 94 °C for 2 min; 28 cycles of 94 °C for 30 s, 57 °C for 1 min, and 72 °C for 1 min; and a final extension cycle of 10 min at 72 °C; and *dmdA*, 94 °C for 2 min; 35 cycles of 94 °C for 20 s, 42 °C for 30 s, and 68 °C for 30 s; and a final extension cycle of 5 min at 68 °C. Though we successfully detected the presence of these amplicon products, the amplicons were not sequenced and validated through cloning and sequencing. As such, this approach was used as a proxy for selection of pBMCs. A total of 19 bacterial isolates were selected based on positive amplification signals for one of the three functional genes, and the nearly full-length 16 S rRNA gene was PCR-amplified from the genomic DNA samples, using the primer set 27 f (5′-AGA GTT TGA TCA TGG CTC AG-3′) and 1492r (5′-GTT TAC CTT GTT ACG ACT T-3′) [47]. The PCR was performed using 5 µL of 10X buffer, 2.0 mM MgCl_2_, 0.2 mM dNTPs, 5 mM of each primer, ca. 2–4 ng of genomic DNA, and 2.5 U Taq DNA polymerase (Promega), in a final volume of 50 µL. The thermal-cycling protocol was as follows: 94 °C for 3 min; 35 cycles of 94 °C for 40 s, 55 °C for 1 min, and 72 °C for 2 min; and a final extension cycle of 10 min at 72 °C. The PCR products were purified using the GFX PCR DNA and Gel Band Purification kit (GE Healthcare, Little Chalfont, UK) and then sequenced (Macrogen Inc., Seoul, South Korea) using the primers 27 f, 1492r, 532 (5′-CGT GCC AGC AGC CGC GGT AA-3′) and 907 (5′-CCG TCA ATT CMT TTG AGT TT-3′) to provide the nearly full-length 16 S rRNA gene sequence of each isolate [47]. The sequencing electropherograms were processed using The Ribosomal Database Project II (RDP) [48] to remove low-quality bases. Sequences of each isolate were assembled into contigs using Bioedit 7.0.5.3 [49]. The bacterial 16 S rRNA gene sequences were analyzed using BLASTn [50]. All sequences were deposited in the NCBI database under accession number SUB3733320.

### Preparation of the BMC consortium

Five bacterial strains isolated from coral tissue and two from the surrounding water were chosen to compose the pBMC consortium. The selection was based on the presence of pre-defined genetic and/or phenotypic characteristics (Supplementary Table S1). The cell number of each individual pBMC grown was estimated using optical-density spectrophotometer (OD_600_) (UV-1800 Spectrophotometer, Shimadzu, Kyoto, Japan) measurements for cultures grown at 26 °C in 100 mL of MB medium for 6, 12, 20, 28, 38, and 48 h, and correlated directly with the number of colony-forming units (CFUs) of each strain at each time point. The CFU numbers were assessed using serial dilutions (10^–4^, 10^–5^, and 10^–6^) of replicates (*n* = 3) of each strain, which were plated in triplicate on MA. A volume of 10 μL of each dilution was applied to MA plates, and colonies were counted with the aid of a colony counter [51], at each collection time point (i.e., 6, 12, 20, 28, 38, and 48 h). Results were normalized to the volume of 1 mL of medium to estimate the cell number at each sampling time. 28 h after inoculation in 100 mL of MB medium, all strains had reached 3.5 × 10^6^ viable cells mL^–1^. Cultures were centrifuged at 5000*g* for 2 min and cell pellets were washed 3 times in NaCl (0.85%), followed by centrifugation at 5000*g* for 2 min, and re-suspended in 30 mL of NaCl (0.85%), to reach the concentration of 10^7^ viable cells mL^–1^. At this stage, CFU counts were performed (as previously described), to confirm that all strains were at 10^7^ viable cells mL^–1^. Also, an antagonism test (as previously described) was performed for all strains, to exclude isolates that showed antagonistic activity against each other within the selected pBMC strains. Equal volumes of 30 mL of pBMC-NaCl (0.85%) solution of each isolate were mixed, resulting in a total volume of 210 mL pBMC-NaCl (0.85%) solution containing 10^7^ cells mL^–1^ of each isolate.

### Mesocosm experimental design

**Fig. 1:**
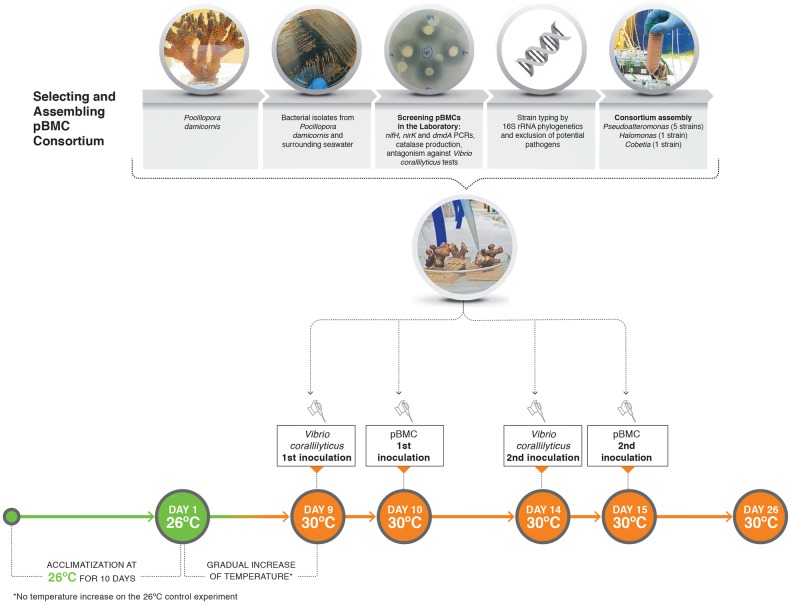
Flowchart showing the experiment overview. The bacterial isolates screened for BMC features were obtained from *Pocillopora damicronis* nubbins and surrounding water, then selected and assembled as a consortium. The following treatments were tested: *Vibrio* (*n* = 3), pBMC + VC (*n* = 3), control (no pBMC or *Vibrio corallilyticus* inoculation) (*n* = 3) and pBMC (*n* = 3), at 30 °C. The aquariums with different treatments were randomly distributed. A parallel set of the same experiment was performed at 26 °C, as a control, where the temperature was not raised at any time

The experimental set-up encompassed 24 individual 1.3-L aquariums, placed in water baths to maintain temperature. Three *P. damicornis* colonies that had been maintained in the original aquaria for ~8 months and for 60 days in the experimental tanks were fragmented into smaller coral nubbins (ca. 5 cm) representing sampling units, i.e., single fragments for each treatment and sampling time. Each treatment was represented by 3 individual aquariums and each aquarium contained 3 coral nubbins (one for each sampling time), and treatments were randomly distributed among water baths (Fig. 1). Each aquarium had individual 26 L circulation sumps to form a circulating loop (Supplementary Fig. S1). The water circulation between aquarium/sump was performed by a water pump (Mini A, Sarlo Better, São Caetano do Sul, Brazil). Importantly, there was no water exchange between/among aquariums and water baths. The aquariums were supplied with artificial seawater (OceanTech, Porto Alegre, Brazil). The seawater flow rate between the aquarium and each individual 26-L sump was 250 mL min^–1^ for each aquarium, providing a tenfold replacement of the volume every hour. In addition, partial exchanges of 20% of the sumps were made every 3 days. The aquariums received only natural sunlight and therefore followed the natural day/night cycles; the experiment was conducted during the austral spring, with sunrise at ca. 06:00 and sunset at ca. 19:00 h. The experiment was covered with a 70% shade screen, resulting in 250 µmol photons m^–2^ s^–1^ at noon, which is consistent with the average parameters measured in the coral donor aquariums. One 1,000 L water tank (master tank), which was kept at 18 °C and interconnected with all four water baths, was used for cooling when necessary. Water pumps (Better 2000, Sarlo Better) in the master tank fed the cold water to the water baths, and the water was returned through two holes in the side of each water bath (Supplementary Fig. S1). Heating (when necessary) was achieved by using six 100 W heaters (Atman, China) in each water bath. The water in each water bath was circulated and mixed by two aquarium pumps (SB 1000 A, Sarlo Better) to maintain homogeneous temperatures. The temperature of the water baths was controlled with Full Gauge controls MT-518ri (Canoas, Brazil) connected to heaters and pumps that fed the cooled water from the master tank to the water baths. The temperature of each aquarium was also measured twice daily. Physical and chemical parameters of the water, including pH, salinity, and dissolved oxygen (DO) were measured every 2 days (Supplementary Table S2).

### BMC mesocosm experiment

Two temperature regimes (26 °C and 30 °C) and 4 treatments were compared in the mesocosm experiment: (i) control samples (CTR) without inoculation of pBMC or VC; (ii) VC, pathogen inoculation (10^5^ cells of *Vibrio coralliilyticus*, at days 9 and 14); (iii) pBMC, consortium inoculation (10^7^ cells of the pBMC consortium, at days 10 and 15); and (iv) pBMC + VC, pathogen (10^5^ cells of *Vibrio coralliilyticus* inoculated at days 9 and 14) and consortium (10^7^ cells of the pBMC consortium inoculated at days 10 and 15).

Following the initial 10-day coral acclimation period at 26 °C, in four of the treatments, the temperature was gradually increased from day 1 (22/10/2016) to day 9 (30/10/2016) until it reached 30 °C. As the peak temperature was reached on day 9, this day was used as the starting point for the manipulation experiment (Fig. 1). The experiment was carried out for a total of 26 days. In a parallel experiment, the same treatments were maintained at 26 °C until day 26. Samples were taken from control aquariums at days 1 and 9 (at this time-point all aquariums were controls, i.e. before VC or pBMC inoculation), and from all treatments on day 26. The protocol used for the inoculation of the pBMC consortium consisted of removing the coral fragments from the aquarium, placing them in a sterile Petri dish, and immediately inoculating 1 mL of the pBMC-NaCl (0.85%) solution, consisting of 1 × 10^7^ cells of the pBMC consortium, or 1 × 10^5^ cells of the VC YB-NaCl (0.85%) solution directly onto the coral fragments. After inoculation, the fragments were carefully returned to the aquariums and the individual Petri dishes were rinsed into the aquarium water. The control fragments were treated in a similar manner, using a sterile saline solution. VC YB was inoculated at days 9 and 14, and the pBMC consortium was inoculated at days 10 and 15 (Fig. 1). The pBMC consortium was inoculated a day after VC to provide an ‘advantage’ to the pathogen in the system. In doing so, assess if the pBMC treatment is effective, even when the pathogen is present at high abundance.

### Quantification of parameters associated with coral health

The color of the coral tissues was analyzed using standard photographs taken on days 1, 9, and 26 of the experiment. Photographs were analyzed in comparison to the Coral Color Reference Chart [52, 53]. For each photograph, the mean gray value of the chart squares was measured (in triplicate) using Photoshop CC 2015, and a standard color curve was created. The mean gray value was calculated for each coral fragment per time point, and the standard curve was used to relate the gray value to a color score. Changes in color per time point were calculated as the final color value minus the initial value. Statistical differences of changes in color analyses were determined using Student’s *t*-test.

The photosynthetic efficiency of *Symbiodinium* was assessed using pulse-amplitude-modulated (PAM) fluorometry as a proxy for coral holobiont health [54]. We used a submersible diving-PAM system (Walz GmbH, Effeltrich, Germany) fitted with a red-emitting diode (LED, peak at 650 nm) and an 8-mm standard glass fiber-optic probe, which was positioned above the oral disk of the polyps. To avoid interference from diurnal photo-inhibition artefacts, measurements were taken after sunset to ensure full recovery of the reaction centers. The maximum quantum yield of PSII photochemistry was determined as *F*_*v*_/*F*_*m*_, where *Fv* was obtained as *Fm–Fo*. *Fo* is the initial fluorescence signal detected under the modulated measuring light of the PAM (weak pulsed light <1 µmol photons m^–2^ s^–1^) and *Fm* is the maximum fluorescence level detected using a short saturating pulse of actinic light. The diving-PAM was configured as follows: Measuring Light Intensity (MI) = 5, Saturation Pulse Intensity (SI) = 8, Saturation Pulse Width (SW) = 0.8, Gain (G) = 3, and Damping (D) = 2. Throughout the experiment, the same coral nubbin from each replicate (*n* = 3 nubbins) was used to measure chlorophyll fluorescence at different sampling times. Statistical differences of *F*_*v*_/*F*_*m*_ analyses were determined using analysis of variance (ANOVA) with STATISTICA 10 software (StatSoft, Tulsa, OK, USA) followed by a Tukey post-hoc test.

### Coral microbiome data analyses

One entire *P. damicornis* coral nubbin was removed from each aquarium (3 aquariums per treatment) at each sampling time, and macerated in a mortar under dry conditions, using a pestle. Total DNA was extracted from 0.5 g of the macerated tissue, using the Qiagen DNAeasy Power Soil kit (Qiagen, Hilden, Germany). The DNA concentration was determined using a Qubit fluorometer (Invitrogen) and subsequently stored at −80 °C.

Quantifications of the bacterial 16 S rRNA genes (for total bacteria) and *dnaJ* genes (used as a proxy for the abundance of VC) [55] were obtained using quantitative PCR (qPCR). The bacterial 16 S rRNA gene assay was based on the SYBR-green chemistry, with reactions performed in an ABI Prism 7300 Cycler (Applied Biosystems, Darmstadt, Germany) in 20 µL reactions containing 10 µL GoTaq qPCR Master Mix 2 × (Promega), 2 µL of DNA template (standardized to 20 ng) and 0.25 mM each of primers 357 f and 529r [56], supplemented with 0.5 µL of 20 mg mL^–1^ of BSA. PCR conditions consisted of an initial denaturation step of 95 °C for 5 min, 35 cycles of 95 °C for 1 min, 57 °C for 1 min, and 72 °C for 45 s [57]. For estimated *dnaJ* gene abundances, the assay consisted of 10 μL GoTaq qPCR Master Mix 2 × (Promega), and 0.25 mM each of primers Vc_dnaJ_F1 and Vc_dnaJ_R1 [55]. The amplification reactions were performed with an initial denaturation step at 94 °C for 3 min, followed by 40 cycles of 95 °C for 15 s (denaturation) and 60 °C for 60 s (annealing/extension). All samples were quantified in triplicate reactions, and sterile H_2_O was used as negative controls. Gene abundances were inferred for each sample, based on standard curves constructed using known concentrations of plasmid DNA extracted from a small fragment of *Escherichia coli* 16 S rRNA gene (357–529 bp) inserted into a pGEM®-T Easy Vector System (Promega), and grown in *E. coli* JM109 strain. The specificity of the amplification was confirmed by melting-curve analysis. Statistical differences of qPCR analyses were determined using analysis of variance (ANOVA) followed by a Tukey post-hoc test.

The V4 variable region of the bacterial 16 S rRNA gene was amplified using the primer set 515 F/806 R. Paired-end (2 × 250 bp) sequencing was carried out at the Argonne National Laboratory (Lemont, IL, USA) in the Next Generation Sequencing Core on an Illumina Miseq. A total of 3,134,212 sequences ranging from 27,632 to 173,206 pairs of reads per sample (average of 76,444 pairs of reads per sample) were obtained from two sequencing runs. Raw reads were trimmed to 240 base pairs for the forward reads and 160 for the reverse reads, allowing for a maximum of 2 errors per mate, no ambiguous bases, and a Qscore ≥20. A total of 2,022,620 reads passed quality control, with an average of 49,332 reads per sample. By using the DADA2 tutorial, after error learning, dereplication, read merging and chimera removal, we obtained a total of 3214 unique features that were subsequently filtered for Bacterial taxa and against Mitochondria and Chloroplast (Phyloseq 1.22.3). The taxonomy was assigned using DADA2 and the SILVA v128 dataset. Additional taxonomy matching was required to filter out features mapping to the host coral mitochondria. Blastn (version 2.6.0 + ) was used to match the features to the reference sequences (EF526303.1 and EU400214.1) using default values. The features discarded had a 97.9% match or better. Prior to statistical analyses, samples were rarified to an equal depth of 1716 sequences per sample (the fewest in a single sample) to minimize effects of sequence depth. Shannon index and ASVs counts were determined using the R package phyloseq [58]. All scripts and data are available in the NCBI Sequence Read Archive (SRA) under accession number PRJNA436030.

Statistical analyses were carried out in two steps. First, differences in bacterial community composition (β-diversity) were calculated. To this end, Bray–Curtis similarities were calculated based on rarefied and square-root-transformed ASV abundances. Permutational multivariate analysis of variance (PERMANOVA) [59] was performed using the homonymous routines in PRIMER6 + [60]. PERMANOVA main test and pair-wise tests were performed using treatment as the main factor, allowing for a full permutation of the raw data with Monte-Carlo tests accounting for type III error, where the fixed effects sum to zero with 10^3^ permutations. Inter-sample Bray-Curtis distances (i.e. pairwise distances between replicates) are shown as boxplots (Supplementary Fig. S2). Second, amplicon sequence variants (ASVs) that could be used to classify the different treatments with accuracy were determined. Hereafter, these ASVs are termed bioindicators. These were determined using a three-step approach. First, we selected ASVs that were statistically significant for (a) the controls, (b) samples collected 26 days after the beginning of the experiment incubated at 26 °C, and (c) samples collected 26 days after the beginning of the experiment incubated at 30 °C using a two-way ANOVA. To account for multiple comparisons testing, we used the false discovery rate (FDR) method available in the “lsmeans” R package. Second, we used a classification based on random forest analysis [61] to evaluate if estimated ASVs classified samples according to their specific treatments. Third, we identified the intersection between the ASVs that were statistically significant and the ones that were most relevant for the random forest classification.

## Results

### pBMC consortium selection and assembly

A total of 30 bacterial isolates were recovered from macerated *P. damicornis* fragments, and 23 from the surrounding seawater. These isolates were screened for potential BMC genes and traits. A total of 44 isolates showed detectable catalase production activity, although only one (*Pseudoalteromonas* sp.-affiliated strain; 16 S rRNA gene 100% identity) (Supplementary Table S1) had antagonistic activity against the coral pathogen *V. coralliilyticus* (strain YB; DSM19607). Six isolates were positive for PCR amplification of the *nif*H gene subunit, 4 for the *nir*K gene subunit, and 1 for the *dmd*A gene subunit (Supplementary Table S1). A total of 35 isolates were affiliated with *Vibrio* spp., 14 with *Pseudoalteromonas* sp., 2 with *Halomonas* sp., and 2 with *Cobetia* sp. (genera *Halomonas* and *Cobetia* are members of the family Halomonadaceae). All *Vibrio* sp. isolates were excluded, considering their pathogenic potential to corals [62, 63], and a pBMC consortium was established from the remaining strains. The selection was based on the diversity of genera with morphologically different visual features, in addition to the presence of one or more of the screened pBMC features. Based on this criteria, 5 morphologically different *Pseudoalteromonas* sp. (out of 14 isolates) obtained from coral tissue were selected, in addition to one representative of each of the other 2 bacterial genera (*Cobetia* and *Halomonas*), both obtained from seawater. The *Pseudoalteromonas* strains displayed rapid growth during the first 6 h of culture, though all strains in the pBMC consortium displayed similar viable counts, i.e., reaching ca. 1 × 10^7^ cells mL^–1^ (Supplementary Fig. S3) after 20 h, and a lag phase at 28 h.

### pBMC mitigates coral bleaching signs from thermal and pathogen stresses

**Fig. 2:**
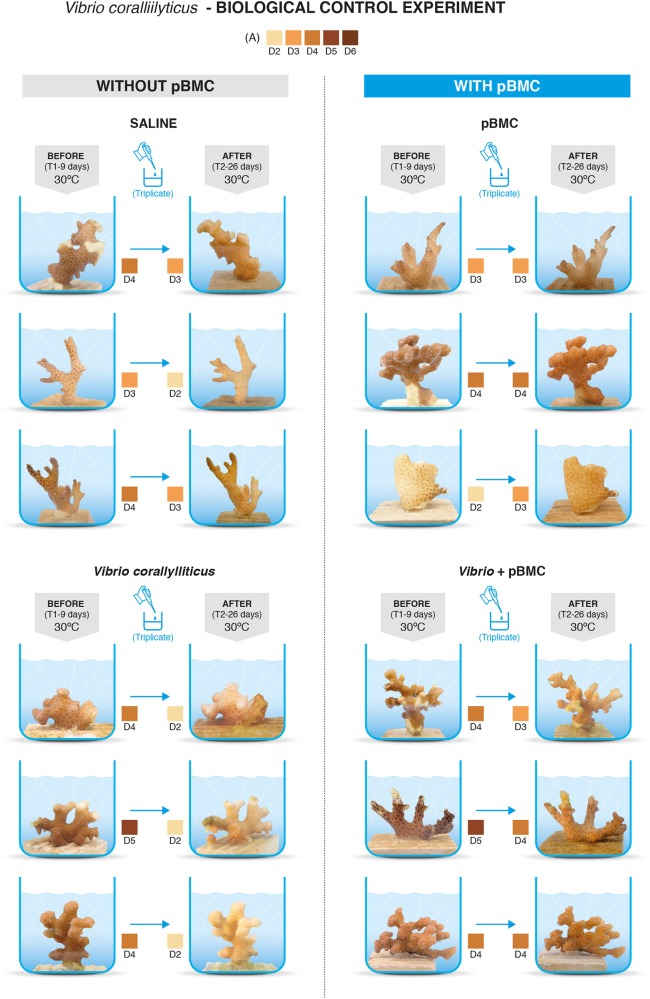
Comparative photos of the *Pocillopora damicornis* fragments at the beginning and at the end of the biological control experiment used in the following 4 treatments: saline (control (CTR), no pBMC inoculation) (*n* = 3), pBMC (pBMC inoculation) (*n* = 3), *Vibrio coralliilyticus* (VC inoculation), pBMC + VC (pBMC and VC inoculation). “Before” corresponds to each experiment initial time (Day 9 of experiment corresponds to the initial time control, i.e., peak of temperature and first inoculations were made or started at day 9). “After” corresponds to the end of the experiment (day 26). *original photographs are shown

The visual appearance of *P. damicornis* coral nubbins, assessed using the Coral Health Chart [53], demonstrated that all treatments maintained at 26 °C displayed no visible color pigment shift during the 26 days of the experiment. In contrast, all replicates of the CTR treatment raised to 30 °C (thermal stress) displayed characteristic signs of stress, with a decrease of 1 unit based on the Coral Health Chart [52]. One replicate of the pBMC treatment at 30 °C also showed a 1-unit decrease, although no visual shifts were detected in other replicates. The VC*-*challenged treatment (without pBMC addition) at 30 °C showed clear signs of bleaching, with decreases of 2–3 units for all replicates (Fig. 2). For the pBMC + VC-challenged coral treatments, however, only one replicate displayed a small 1-unit decrease, while the remaining replicates showed no signs of bleaching. Regarding the visual shifts observed between treatments at 30 °C, significant differences were detected between those treated with VC compared to those treated with pBMC, between corals treated with VC and controls, and between corals treated with VC compared to corals treated with pBMC + VC (Student’s *t*-test: *P* < 0.05) (Supplementary Table S3).

**Fig. 3:**
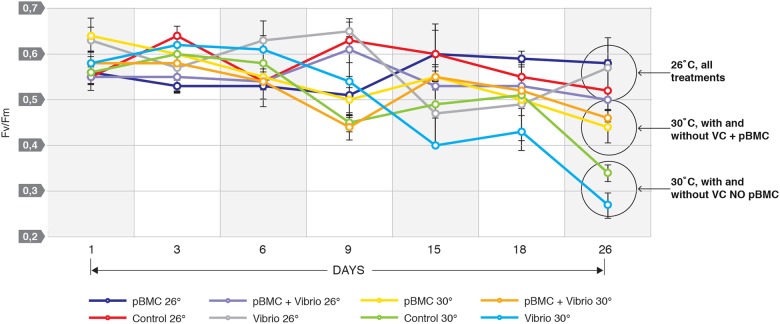
Measurements of *F*_*v*_/*F*_*m*_ in *Pocillopora damicornis* at 30 °C and 26 °C during 26 days of experiment, with the following treatments: control, no inoculation (CTR), pBMC (pBMC consortium inoculation), VC (*Vibrio coralliilyticus* inoculation), pBMC + VC (pBMC consortium and *Vibrio coralliilyticus* inoculation) control (CTR) (*n* = 3)

Results from the visual response of the coral nubbins across each treatment were confirmed by PAM fluorometry measurements that calculated the *F*_*v*_/*F*_*m*_ values indicative of *Symbiodinium* photosystem function. For corals maintained at 26 °C, including the CTR and pBMC treatments, the *F*_*v*_/*F*_*m*_ values were similar, ranging between 0.50 and 0.60 (Fig. 3) and did not present significant differences (*P* > 0.05). In contrast, all treatments at 30 °C showed reductions in the *F*_*v*_/*F*_*m*_ ratios, with a decrease in the *F*_*v*_/*F*_*m*_ ratios of 54, 40, 31, and 20% respectively for treatments VC, CTR, pBMC, and pBMC + VC, when compared directly for days 1 and day 26. Similarly reduction of *F*_*v*_/*F*_*m*_ ratios of 50, 24, and 12% for VC, CTR, and pBMC respectively were observed when comparing day 9 (temperature peak and VC inoculation) to day 26 (the end of the experiment) (Fig. 3). Interestingly, the coral nubbins challenged with VC but also inoculated with pBMC (pBMC + VC), at 30 °C, displayed a 6% increase in the *F*_*v*_/*F*_*m*_ ratios between day 9 and 26 (Fig. 3), a 56% higher mean compared to the treatment exposed solely to VC (ANOVA: F: 14,15; df = 1; *P* < 0.05) at 30 °C. Significant differences were also observed in comparisons between other treatments at 30 °C, such as higher *F*_*v*_/*F*_*m*_ ratios in pBMC corals compared with VC (ANOVA: F: 27,69; df = 1; *P* < 0.01), pBMC compared with thermal-stressed controls (ANOVA: F: 8,96; df = 1; *P* < 0.05), and pBMC + VC against thermal-stressed controls (ANOVA: F: 8,43; df = 1; *P* < 0.05).

At 30 °C, the abundance of the bacterial 16 S rRNA gene copies peaked after 26 days for pBMC (1.48 × 10^2^–3.26 × 10^3^ gene copies mL^–1^), VC (2.10–4.06 × 10^2^ gene copies mL^–1^), and pBMC + VC (3.84 × 10^2^–9.49 × 10^3^ gene copies mL^–1^) treatments, and there were no significant differences among these treatments (*P* > 0.05). However, when each treatment was compared to the CTR (4.62–8.88 × 10^1^ gene copies mL^–1^; *P* *<* 0.01), at 26 days, significant differences were observed (*P* *<* 0.01). In addition, copies of the *dnaJ* gene, specific for vibrios, were detected only in the VC treatment (6.48 × 10^1^–1.42 × 10^4^ gene copies mL^–1^) at 30 °C, but not in the CTR (no addition of VC and/or pBMC) and corals inoculated with pBMC + VC or any other treatment at 26 °C.

**Fig. 4:**
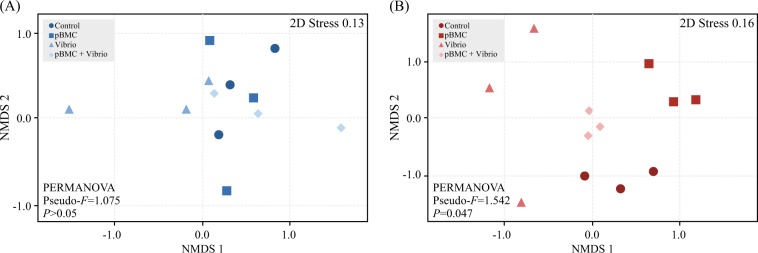
NMDS plots of Pocillopora damicornis microbiome at 26 °C (**a**) and 30 °C (**b**), at day 26, based on high-throughput sequencing data (*n* = 3). Statistics are provided as inset panels

Bacterial community profiling of coral nubbins identified diverse communities associated with the experimental coral fragments, although no significant differences in alpha-diversity metrics (ASV counts and Shannon index) were observed across most of the treatments (pairwise *t*-test, *P* > 0.05, Supplementary Fig. S4). The exception was observed for the pBMC-treatment samples collected after 26 days of incubation at 30 °C, which displayed ASV counts lower than the corresponding pBMC + VC treatment (pairwise *t*-test, *P* *<* 0.05, Supplementary Fig. S4). In addition, no clear separation of treatments was observed when comparing all samples from the different temperatures collected 26 days after the beginning of the experiment (Supplementary Fig. S5). To confirm these results, no treatment pair-wise statistical differences were detected (PERMANOVA, 10^3^ permutations, *P* *>* 0.05) across treatments and between samples incubated at 26 °C or 30 °C (Supplementary Table S5). Therefore, intra-sample variability NMDS plots were generated separately for corals incubated for 26 days at 26 °C or 30 °C (Fig. 4). For samples from day 26 at 26 °C (Fig. 4a), no significant differences were observed between the distances from samples inoculated with pBMC + VC or only with VC (PERMANOVA main test; Pseudo-*F* *=* 1.075, *P* *>* 0.05, see also Supplementary Table S6). At 30 °C, a significant difference across treatments was observed 26 days after the beginning of the experiment (PERMANOVA main test; Pseudo-*F* *=* 1.542, *P* *=* 0.047, see also Supplementary Table S6). In addition, for treatments at 30 °C, pBMC + VC and VC showed significantly (*P* *<* 0.05) lower and higher inter-sample dissimilarities, respectively, when compared with control and pBMC treatments (Supplementary Fig. S2). No significant clustering of replicate samples (*P* > 0.28, Supplementary Table S4) was observed for treatment or sampling time (days 1, 9, and 26) for corals that were not inoculated with pBMC and/or VC at 26 °C or 30 °C (Supplementary Fig. S6). In contrast, for samples incubated for 26 days at 26 °C (Supplementary Fig. S7; *P* > 0.13; Supplementary Table S6) and at 30 °C (Supplementary Fig. S8; *P* < 0.05; Supplementary Table S6), a clear clustering of replicates from pBMC- and pBMC + VC-inoculated corals was observed by Bray-Curtis distances.

**Fig. 5:**
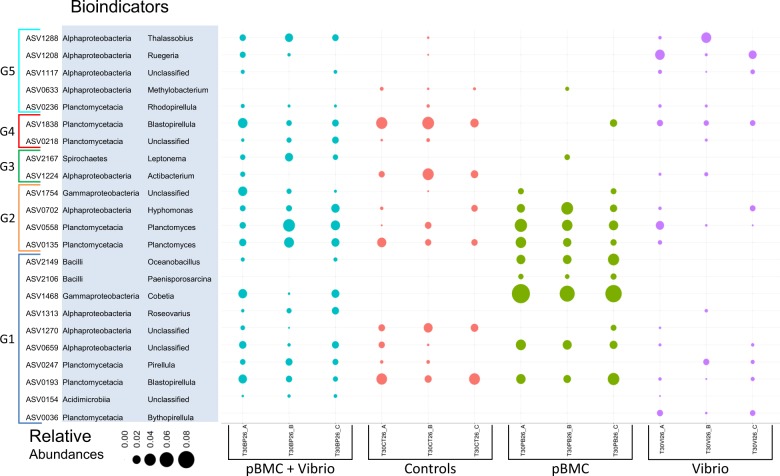
Relative abundance distribution of ASVs used as bioindicators in the different treatments (Controls, pBMC, pBMC + VC and VC) per sample. The size of the circles represent the relative abundances. We added colors to ASV relative abundances belonging to the same treatments. Class and genus of each ASVs are also shown in this figure. We grouped the ASVs in 5 different groups depending on how the different ASVs showed statistic differences (*P* < 0.5) in a False Discovery Rate test performed after a two way ANOVA using inoculation of pBMC and VC as factors. (G1) Statistically significant ASVs (*P* < 0.05) in the interaction pBMC:VC. (G2) Statistically significant ASVs (*P* < 0.05) in the interaction pBMC * (VC * pBMC:VC). (G3) Statistically significant ASVs (*P* < 0.05) in the interaction pBMC:VC * VC. (G4) Statistically significant ASVs (*P* < 0.05) in the interaction pBMC:VC * (VC * pBMC). (G5) Statistically significant ASVs (*P* < 0.05) in the interaction VC * (pBMC * pBMC:VC)

To determine which ASVs correlated with the partial mitigation of bleaching signs for corals at the end of the 30 °C experiment, the ASVs that were significantly different in at least one of the treatments were detected in a 2-way ANOVA and *P*-values adjusted using the false discovery rate method, where inoculations of pBMC and VC were used as factors. A total of 30 SVs were significantly different in at least one treatment (*P* *<* 0.05) (Supplementary Fig. S8, Supplementary Table S7). In contrast, only 10 ASVs were significant in at least one treatment for samples incubated at 26 °C after 26 days of the experiment (*P* *<* 0.05) (Supplementary Fig. S7, Supplementary Table S8). The results of a two-way ANOVA with multiple correction testing for each sample group (control, 26 °C and 30 °C) under different treatments are shown in Supplementary tables S9, S10, and S11, respectively. Further, our random forest analyze results demonstrated that, at the end of the experiment (day 26) samples incubated at 30 °C with 0% error, while ~42% of classification errors were detected for samples incubated at 26 °C (Supplementary Table S12). The mean decrease Gini score obtained in the random forest analysis for samples incubated at 30 °C after 26 days of the experiment showed that 24 ASVs had considerably higher scores than all others for classifying the different treatments with accuracy (Supplementary Fig. S9). To determine the bioindicators, we intersected the 30 ASVs that were statistically significant and 24 most relevant for the classification of samples after 26 days of the experiment at 30 ^o^C (Fig. 5). A total of 23 ASVs were classified as bioindicators, and further separated into five groups (Fig. 5). From those ASVs that showed significant differences in the interaction between pBMC and pBMC + VC, an ASV classified as *Cobetia* was the most dominant in those samples inoculated only with pBMC, followed by an ASV classified as *Oceanobacillus* (a box plot with relative abundances of these ASVs is shown in Supplementary Fig. S10–12). Most of the ASVs that were classified as bioindicators belonged to Alphaproteobacteria (9 ASVs), followed by Planctomycetes (8 ASVs) (Fig. 5). ASVs belonging to *Ruegeria* and *Thalassobios* were the most dominant ASVs present in the VC treatment (Fig. 5, Supplementary Fig. S10).

## Discussion

Recent assessments have documented declines in coral cover driven by both local and global anthropogenic impacts, including increased frequency of large-scale bleaching events [7, 8]. While the priority actions to protect coral-reef ecosystems should focus on minimizing global changes and CO_2_ emissions [8], other palliative actions are required to stem or even reverse these declines. Currently, there is no feasible treatment that can minimize temperature bleaching and/or disease impact on corals in the field at reef scale. Current research efforts have been focused on restoring local populations through propagation and gardening approaches or selecting resistant coral communities (i.e., assisted breeding, assisted translocation, and human-assisted evolution) [11, 13, 64]. The development of protective management or treatments to be applied in situ, to build or enhance the resistance and/or resilience of corals in the field represents a useful complementary strategy.

This study presents the first attempt to extrapolate the well-established use of bacterial consortia [65–67] to protect or improve the health of corals. It should be noted that this experimental study used artificial seawater in aquaria, which may influence the coral microbiomes when compared to those in natural settings. The results generated from a controlled and replicated aquarium experiment demonstrated the ability of a selected pBMC consortium to partially mitigate coral bleaching induced through both temperature and putative pathogen challenge, proving the concepts that (a) it is possible to manipulate the coral microbiome and (b) this manipulation can influence the coral health status. The inoculated pBMC consortium increased coral resistance to bleaching, as assessed through visual pigment levels and *Symbiodinium* photochemical efficiency. These improvements in coral-bleaching metrics were observed in pBMC-inoculated corals at 30 °C, in contrast to controls without pBMC addition, which displayed strong bleaching signs, as indicated by significantly lower photopigment contents and *F*_*v*_/*F*_*m*_ ratios. Even for corals challenged with the pathogen *V. coralliilyticus*, treatments without pBMC addition bleached, while treatments in which the pBMCs were added 1 day after VC challenge displayed no signs of bleaching. This physiological response indicates that the pBMC inoculation partially mitigated the bleaching process, both when corals were subjected to a temperature stress alone and when they were also challenged with a bacterial pathogen. Considering that coral mortality levels caused by bleaching depend on the intensity and extent of the seawater-temperature event and the ability of the holobiont to resist other ancillary challenges such as microbial infections [68], any approach that increases coral resistance represents an important strategy that can benefit coral reef ecosystems.

The quantification of total bacteria and *V. coralliilyticus* abundances in coral nubbins demonstrated that *V. coralliilyticus* was detected only in treatments without the added pBMC consortium. For example, 6.48 × 10^1^–1.42 × 10^4^ *dnaJ* gene copies mL^–1^ were detected from VC-treated corals, while no gene copies were detected in any other treatments, including pBMC + VC, suggesting biological control of VC by the inoculated pBMC consortium. The importance of manipulating the coral microbiome and tracking coral health was recently highlighted by Welsh et al[32]., who demonstrated the predation of *V. coralliilyticus* by *Halobacteriovorax*, and that specific predator(s) can control “alien” coral-opportunistic pathogens. Biocontrol of microbial diseases has been used widely across many environments and for different organisms [69–71], but this approach is still a little-explored concept for coral protection. Our data support the potential usefulness of this approach, by indicating that coral health can be improved through biological control of the inoculated *V. coralliilyticus* pathogen. Different mechanisms are likely to be involved in this process, including the direct antagonistic activity of one or more of the pBMC members, or indirect niche colonization by the pBMCs, which exclude *V. coralliilyticus*. The experimental manipulation may have also promoted shifts in the coral microbiome, thus excluding other opportunistic pathogens or allowing the establishment of other beneficial components, thereby indirectly making corals less vulnerable to bacterial pathogens. Clustering of the coral-associated microbiomes was observed for inoculated corals at 30 °C, and consistent bacterial bioindicators of the different treatments were detected only at 30 °C, indicating that these community shifts were the direct result of the microbiome manipulation in corals under stress, although the exact mechanisms triggering the changes still need to be explored.

The coral microbiomes of the VC treatment at 30 °C, which displayed signs of bleaching, also showed more dispersed community structure between replicates, when compared with control and pBMC-inoculated corals, after 26 days of the experiment. This pattern aligns with the Anna Karenina principle (AKP), proposed by Zaneveld and collaborators [72], in which microbiomes from stressed organisms, in this case, those inoculated with a pathogen, vary more than those associated with “healthy” hosts. The AKP also reinforces the putative benefits that pBMC manipulations may have in influencing the coral host microbiome, i.e., providing greater community stability and therefore minimizing stochastic shifts in the face of stress(es). Following the AKP prediction, it is important to note that coral samples inoculated with VC + pBMC had lower Bray-Curtis dissimilarity than controls or corals inoculated with pBMC. Interestingly, this result aligns with the physiological proxy data, where VC + pBMC samples had higher F*v*/F*m* values than that of pBMC. While tempting to speculate that the inoculation of VC might have favored the establishment of pBMC, for instance through a disturbance-mediated effect, we cannot conclusively confirm this with the data obtained in the current study. Understanding the influence of the pBMC community at the cellular level is an important next step to elucidate this and other questions. It is currently not known if the pBMCs colonize the host and help to establish a healthy microbiome, thereby preventing a random assembly of the community that could be shifted easily by opportunistic and potentially pathogenic members, i.e., the pathobiome [31]. An alternative explanation would consist of pBMCs providing an additional source of nutrition through heterotrophy and stimulated microbial loops that sustain the corals and prevent bleaching, although *Cobetia* sp. being selected as a bioindicator of pBMC treatment at 30 °C suggests that at least this strain was not used primarily as a nutrition source.

This pBMC-inoculated strain, *Cobetia* sp., was a dominant member of the CTR microbiome (26 °C treatment and sampled at days 1, 9, and 26), and a selected bioindicator and high-abundance group correlated with pBMC- and pBMC + VC-inoculated corals at 30 °C. However, *Cobetia*-affiliated sequences were not retrieved from stressed corals (CTR and VC treatments at 30 °C), suggesting that *Cobetia* may not only be sensitive to the applied stress but also have some role in mitigating the temperature and pathogen challenge effects. The bioaugmentation of the surroundings with native pBMCs, as successfully demonstrated by the *Cobetia* inoculation, during events of environmental stress would increase the chances for the uptake and establishment of beneficial (or at least non-pathogenic) microorganisms, instead of pathogen selection and establishment. Once the environmental-stress conditions return to standard conditions, i.e., without the acute selective pressure, the holobiont may return to the original stable state. For example, during a bleaching event, the microbiome of *A. millepora* colonies was shown to shift as the colonies bleached with increasing temperature, although post-bleaching the microbiome returned to a profile similar to the pre-bleaching microbiome [73]. These observations are again consistent with Zaneveld and colleagues [72] in their parallel to Tolstoy’s “War and Peace”.

Other specific bioindicator taxa were consistently selected across treatments incubated at 30 °C, with, for example, the presence of *Ruegeria* sp.-affiliated sequences correlated with VC-inoculated corals. *Ruegeria* species have been previously associated with Yellow Band Disease (YBD), with a consortium of *Vibrio* species implicated in causation [74]. *Roseovarius* sp. and *Thalassobius* sp. groups, both previously associated with coral disease and stress [75–77], were also selected as bioindicators and were detected in VC-inoculated corals only at 30 °C, likely opportunistically taking advantage of compromised coral hosts. In contrast, *Oceanobacillus* sp.-affiliated sequences were associated with pBMC-inoculated corals, with or without concomitant inoculation of VC, while *Paenisporosarcina* sp. were detected only in pBMC-inoculated corals not challenged with VC. The microbial diversity and the relationships between key microbial players may be differently affected by the two stressor agents [78–81]. Ultimately, the beneficial manipulation of key microorganisms may result in a more stable microbiome, which is less vulnerable to microbial invasion [56, 82–84].

This study successfully demonstrated the potential of a pBMC consortium to partially prevent bleaching in temperature- and bacteria-challenged corals over a short-term aquarium experiment. We suggest that this consortium can be considered not merely as  putative BMC consortium (i.e., a pBMC)but as a BMC consortium (without the need for the putative modifier), since a selection of sequences retrieved from our experimental system displayed high identity with the strains incorporated as BMC inoculates (Supplementary Fig. S13). Scaling up our experimental system by including more replicates is required to further validate these findings, and information on the mechanism(s) by which this BMC consortium as a whole, and individual strains, promoted coral resistance is currently lacking. However, at least one of the inoculated strains (*Cobetia* sp.) was an abundant bioindicator of the BMC inoculation in the coral microbiomes at 30 °C at the end of the experiment, suggesting some level of competitiveness and potential niche establishment. In contrast, *Pseudoaltermonas* sp. and *Halomonas* sp.-affiliated sequences were not detected as bioindicators of the BMC treatments at the end of the experiment, even though the corals were inoculated with 5 strains belonging to the genus *Pseudoalteromonas*, indicating that these strains may not have been able to establish as part of the dominant coral microbiome. It is possible, however, that such strains may have indirect beneficial effects by stimulating other microbiome community members, including the establishment of the *Cobetia marina* strain. There is still much to understand about the ecological intricacies of this system in the context of the existing members of the coral microbiome. To further explore the potential of pBMC and pBMC consortia, new approaches for the selection and inoculation of such consortia into the coral holobiont need to be developed. Future studies should also focus on the omics analyses and in situ visualization, to elucidate the interactions between BMCs and the host and other microbiome partners, and establish the mechanisms by which they improve coral health. Such studies will elucidate if the competitive BMCs provide direct benefits to the corals or if the inoculated strains support other key symbiotic populations within the coral microbiome during adverse environmental conditions. Of critical importance, this study demonstrates that the addition of BMC consortium is a potentially promising approach to improve coral fitness. This approach can also contribute to a better understanding of the symbiotic cellular interactions that are essential for contributing to the resistance and resilience in coral populations faced with increasing environmental pressures.

## Supplementary information

Legends

Supplementary Figure 1

Supplementary Figure 2

Supplementary Figure 3

Supplementary Figure 4

Supplementary Figure 5

Supplementary Figure 6

Supplementary Figure 7

Supplementary Figure 8

Supplementary Figure S9

Supplementary Figure 10

Supplementary Figure 11

Supplementary Figure 12

Supplementary Figure 13

Table S1

Table S2

Table S3

Table S4

Table S5

Table S6

Table S7

Table S8

Table S9

Table S10

Table S11

Table S12
