# Supplementary material for: Marine probiotics: increasing coral resistance to bleaching through microbiome manipulation
Source: ISME J. 2018 Dec 5;13(4):921–36. doi: 10.1038/s41396-018-0323-6 (PMC6461899; doi:10.1038/s41396-018-0323-6)
Supplement: Supplementary file 1 — Legends [file 41396_2018_323_MOESM1_ESM.docx]

**Figures Legends**

**Figure 1.** Flowchart showing the experiment overview. The bacterial isolates screened for BMC features were obtained from *Pocillopora damicronis* nubbins and surrounding water, then selected and assembled as a consortium. The following treatments were tested: *Vibrio* (*n* = 3), pBMC + VC (*n* = 3), control (no pBMC or *Vibrio corallilyticus* inoculation) (*n* = 3) and pBMC (*n* = 3), at 30°C. The aquariums with different treatments were randomly distributed. A parallel set of the same experiment was performed at 26°C, as a control, where the temperature was not raised at any time.

**Figure 2.** Comparative photos of the *Pocillopora damicornis* fragments at the beginning and at the end of the biological control experiment used in the following 4 treatments: saline (control (CTR), no pBMC inoculation) (*n* = 3), pBMC (pBMC inoculation) (*n* = 3), *Vibrio coralliilyticus* (VC inoculation), pBMC + VC (pBMC and VC inoculation). “Before” corresponds to each experiment initial time (Day 9 of experiment corresponds to the initial time control, i.e., peak of temperature and first inoculations were made or started at day 9). “After” corresponds to the end of the experiment (day 26). *original photographs are shown.

**Figure 3.** Measurements of *F_v_*/*F_m_* in *Pocillopora damicornis* at 30°C and 26°C during 26 days of experiment, with the following treatments: control, no inoculation (CTR), pBMC (pBMC consortium inoculation), VC (*Vibrio coralliilyticus* inoculation), pBMC + VC (pBMC consortium and *Vibrio coralliilyticus* inoculation) control (CTR) (*n* = 3).

**Figure 4.** NMDS plots of Pocillopora damicornis microbiome at 26°C (A) and 30°C(B) based on high-throughput sequencing data (*n* = 3). Statistics are provided as inset panels.

**Figure 5.** Relative abundance distribution of ASVs used as bioindicators in the different treatments (Controls, pBMC, pBMC + Vibrio and Vibrio) per sample. The size of the circles represent the relative abundances. We added colors to ASV relative abundances belonging to the same treatments. Class and genus of each ASVs are also shown in this figure. We grouped the ASVs in 5 different groups depending on how the different ASVs showed statistic differences (*P*<0.5) in a False Discovery Rate test performed after a two way ANOVA using inoculation of pBMC and Vibrio as factors. (G1) Statistically significant ASVs (*P*<0.05) in the interaction pBMC:Vibrio. (G2) Statistically significant ASVs (*P*<0.05) in the interaction pBMC * (Vibrio * pBMC:Vibrio). (G3) Statistically significant ASVs (*P*<0.05) in the interaction pBMC:Vibrio * Vibrio. (G4) Statistically significant ASVs (*P*<0.05) in the interaction pBMC:Vibrio * (Vibrio * pBMC). (G5) Statistically significant ASVs (*P*<0.05) in the interaction Vibrio * pBMC:Vibrio. (G5) Statistically significant ASVs (*P*<0.05) in the interaction Vibrio * (pBMC * pBMC:Vibrio).

**Supplementary Figure Legends**

**Supplementary Figure 1.** Schematic view of the experimental system. Sump and main centrifugal pump (1); experimental aquariums (2); water bath (3); polycarbonate cover (4); cold-water reservoir (5); chiller unit (6); independent temperature control (7).

**Supplementary Figure 2.** Boxplot displaying within treatment dissimilarity (Bray-Curtis) for treatments at 26°C (A) and 30°C (B) at the end of the experiment (day 26, 26d). Different letters denote independent Mann-Whitney *U* test results (*P*<0.05).

**Supplementary Figure 3.** Growth curve of the seven selected pBMC bacterial strains.

**Supplementary Figure 4.** Alpha-diversity metrics (ASV counts and Shannon index) of *Pocillopora damicornis* microbiome at 26°C (A) and 30°C (B), at the beginning (day 1, 1d), peak of temperature and just before *Vibrio coralliilyticus* (VC) and pBMC inoculations (day 9, 9d) and at the end of the experiment (day 26, 26d) based on high-throughput sequencing data (*n* = 3). CTR (control, no inoculation); pBMC (pBMC consortium inoculation); VC (*Vibrio coralliilyticus* inoculation); pBMC + VC (pBMC consortium and *Vibrio coralliilytic*us inoculation). Samples followed by the same letters were not statistically different (pairwise *t*-test with non-pooled standard deviations, *P*<0.05, *P*-value adjustment method: holm)

**Supplementary Figure 5.** Non-metric multidimensional scaling (NMDS) based on Bray-Curtis dissimilarities of microbial community composition for all samples collected 26 days after the beginning of the experiment. Colors indicates samples incubated at 26^o^C (blue to light blue) or at 30^o^C (red to pink); while shapes indicate the different treatments.

**Supplementary Figure 6.** Clustering of non-inoculated samples (controls) over time. Clusters were produced based on Euclidean distances between samples produced from ASVs that were different between treatments (*P*<0.05). We determined these statistically different ASVs using a two-way ANOVA with False Discovery Rate method (“lsmeans” R package) for multiple comparison testing (time and temperature were used as factors). Rectangles were used to indicate time and temperature for each sample. We added a heatmap showing the relative abundance of each significantly different ASVs. The color coding for the relative abundance can be seen in the left upper corner of the figure.

**Supplementary Figure 7.** Clustering of samples collected on day 26 and incubated at 26^o^C. Clusters were produced based on Euclidean distances between samples produced from ASVs that were different between treatments (*P*<0.05). We determined these statistically different ASVs using a two way ANOVA with False Discovery Rate method (“lsmeans” R package) for multiple comparison testing (pBMC inoculated samples and Vibrio inoculated samples were used as factors). Rectangles were used to indicate samples inoculated with pBMC and those inoculated with Vibrio. Controls can be seen without squares for pBMC or Vibrio. We added a heatmap showing the relative abundance of each significantly different ASV. The color coding for the relative abundance can be seen in the left upper corner of the figure.

**Supplementary Figure 8.** Clustering of samples collected on day 26 and incubated at 30^o^C. Clusters were produced based on Euclidean distances between samples produced from ASVs that were different between treatments (*P*<0.05). We determined these statistically different ASVs using a three way ANOVA with False Discovery Rate method (“lsmeans” R package) for multiple comparison testing (pBMC inoculated samples and Vibrio inoculated samples were used as factors). Rectangles were used to indicate samples inoculated with pBMC and those inoculated with Vibrio. Controls can be seen without squares for pBMC or Vibrio. We added a heatmap showing the relative abundance of each significantly different ASV. The color coding for the relative abundance can be seen in the left upper corner of the figure.

**Supplementary Figure 9.** Random forests variable importance of ASVs used to classify the different samples based on on all ASV abundance distribution for samples incubated at 30°C after 26 days of the experiment.

**Supplementary Figure 10.**  Significant differences of individual bioindicators relative abundance for Alphaproteobacteria between treatments (Control, pBMC,pBMC + Vibrio and Vibrio) using a two way ANOVA with False Discovery Rate method for multiple comparison testing ( * represents samples significantly different *P*<0.05; **, *P*≤0.01;***, *P*≤0.001).

**Supplementary Figure 11.**  Significant differences of individual bioindicators relative abundance for Gammaproteobacteria between treatments (Control, pBMC,pBMC + Vibrio and Vibrio) using a two way ANOVA with False Discovery Rate method for multiple comparison testing (*represents samples significantly different at **P*<0.05; ***P*≤0.01; ****P*≤0.001).

**Supplementary Figure 12.** Significant differences of individual bioindicators relative abundance for Planctomycetes between treatments (Control, pBMC,pBMC + Vibrio and Vibrio) using a two way ANOVA with False Discovery Rate method for multiple comparison testing (*represents samples significantly different at **P*<0.05; ***P*≤0.01;****P*≤0.001).

**Supplementary Figure 13.** (a) Phylogenetic reconstruction of bacterial 16S rRNA gene sequences retrieved from inoculated pBMC and close-match sequences retrieved from the study system using (Illumina MiSeq) high-throughput sequencing. The tree was made using the Neighbor-joining method and Kimura-2 parameter. There were a total of 250 nucleotide positions in the final dataset, and numbers indicates bootstrap values (500 permutations). Inoculated pBMC are highlighted in the tree.(b) Boxplot displaying relative abundances of ASVs that showed more than 99.5% similarity to pBMC 1-7 in samples collected 26 days after the beginning of the experiment incubated at 26^o^C and 30^o^C.
