# Supplementary figures and images for: Marine probiotics: increasing coral resistance to bleaching through microbiome manipulation
Source: ISME J. 2018 Dec 5;13(4):921–36. doi: 10.1038/s41396-018-0323-6 (PMC6461899; doi:10.1038/s41396-018-0323-6)

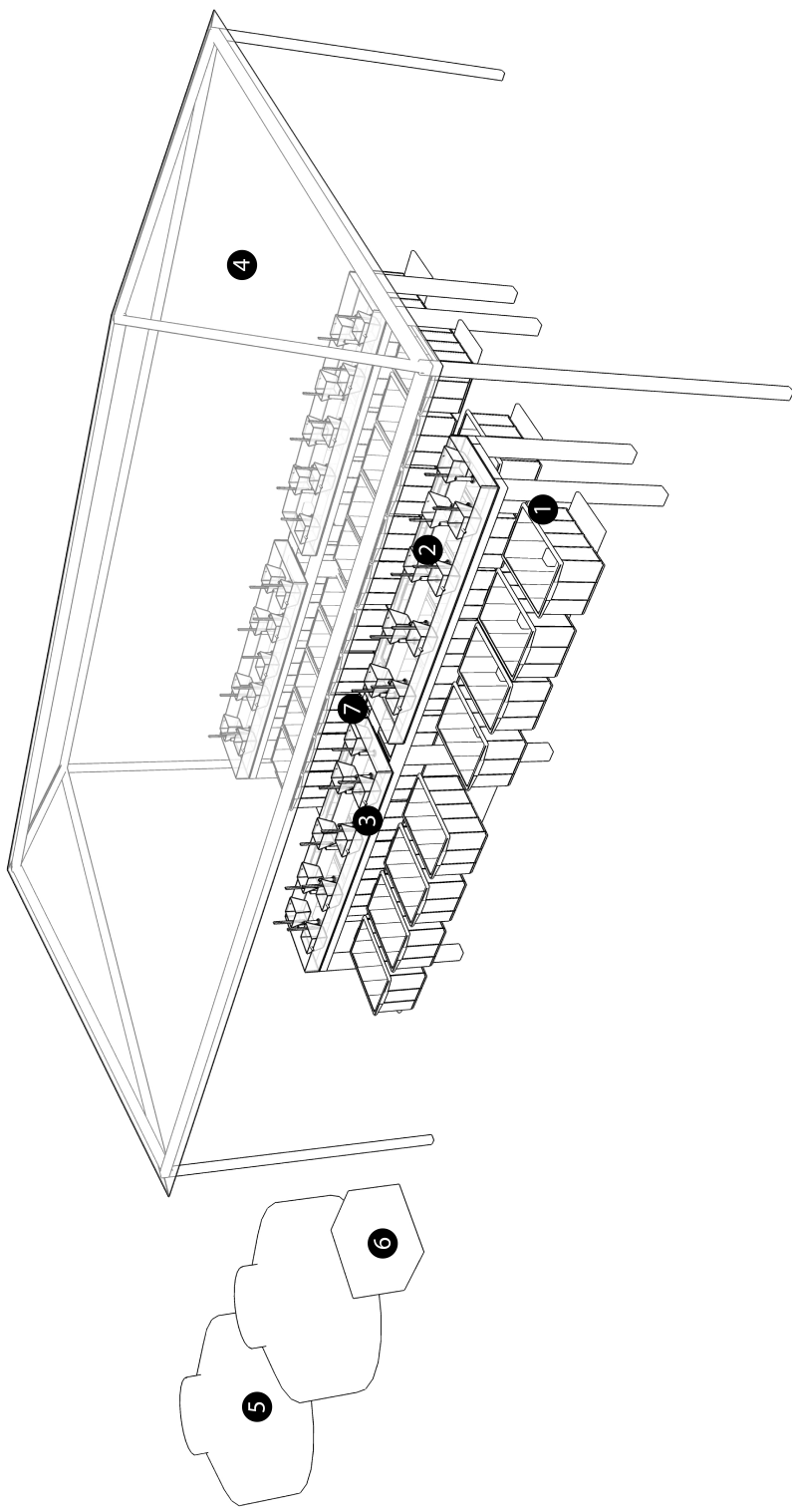

Supplement: Supplementary file 2 — Supplementary Figure 1 [file 41396_2018_323_MOESM2_ESM.pdf]

**Supplementary Figure S2.** Growth curve of the 7 selected pBMC strains.

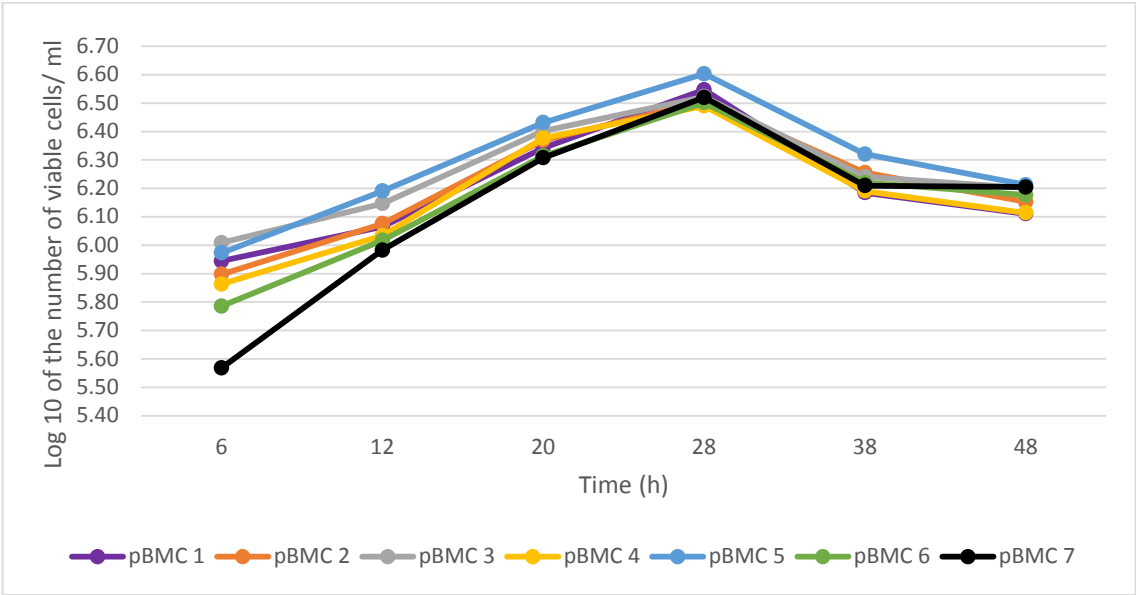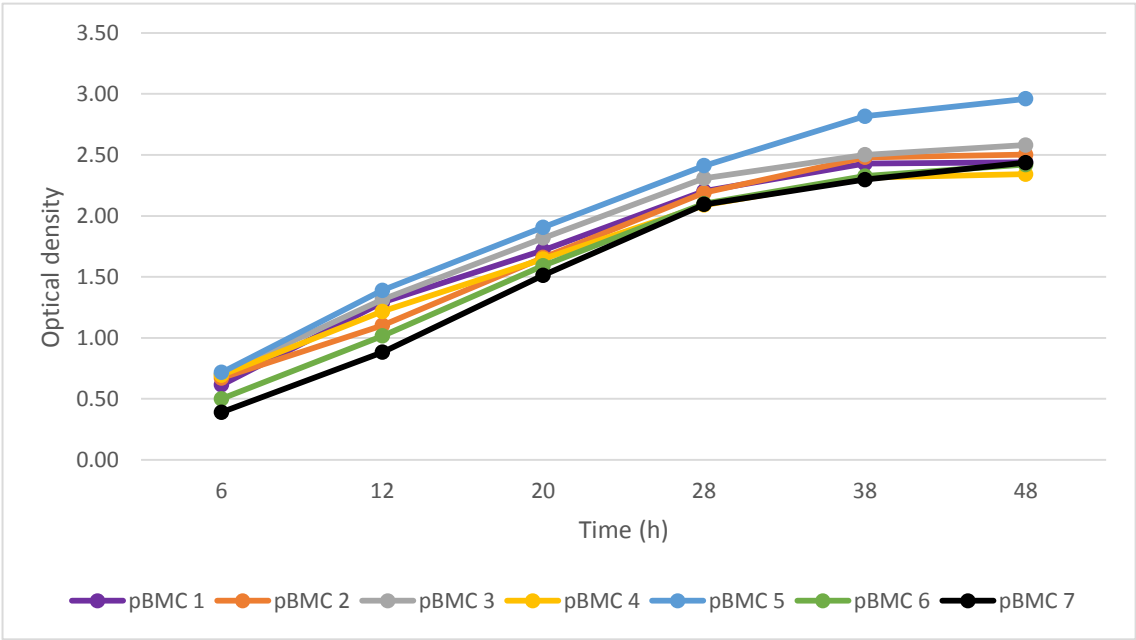

Supplement: Supplementary file 3 — Supplementary Figure 2 [file 41396_2018_323_MOESM3_ESM.pdf]

Amplicon sequence variant (ASV) counts

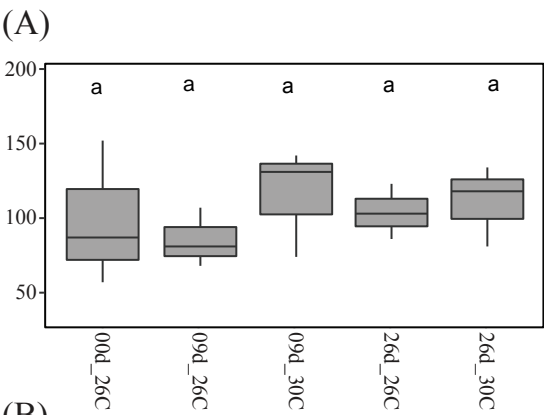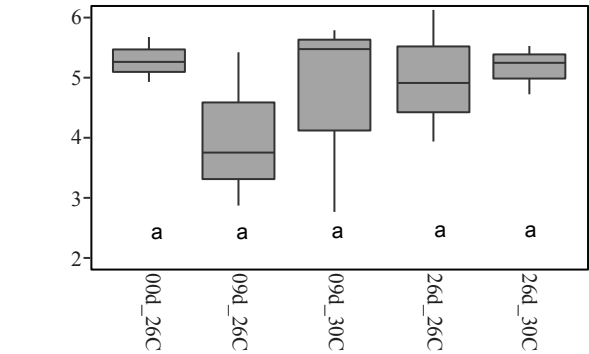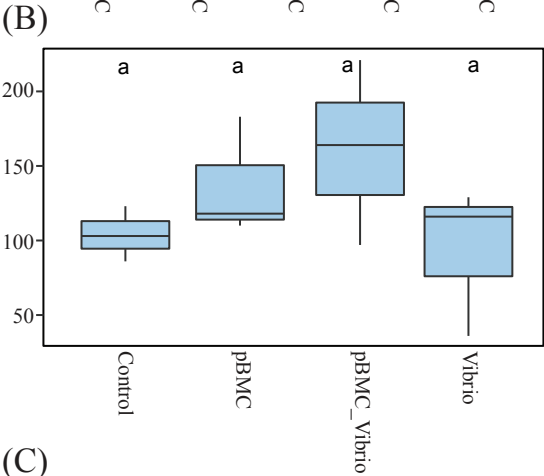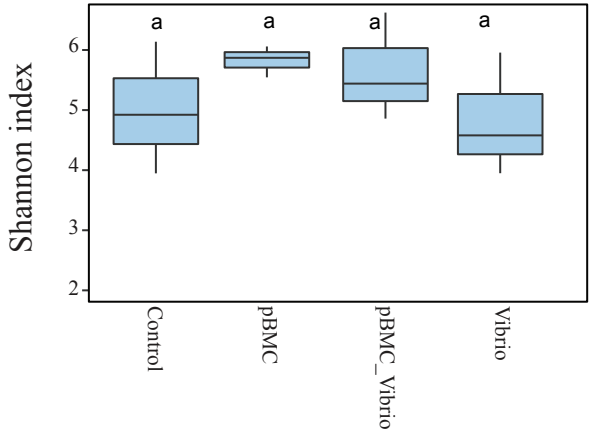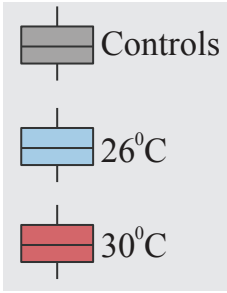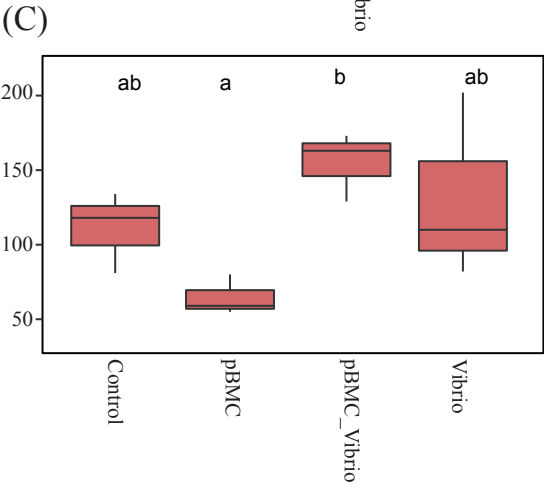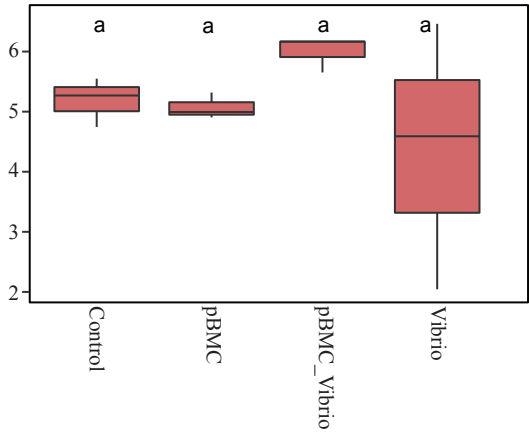

Supplement: Supplementary file 4 — Supplementary Figure 3 [file 41396_2018_323_MOESM4_ESM.pdf]

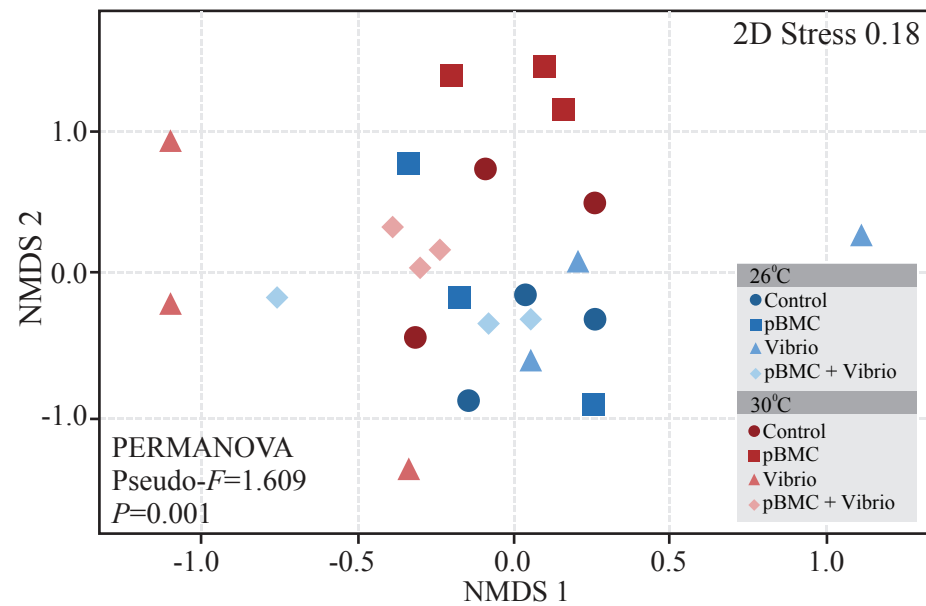

Supplement: Supplementary file 5 — Supplementary Figure 4 [file 41396_2018_323_MOESM5_ESM.pdf]

(A)

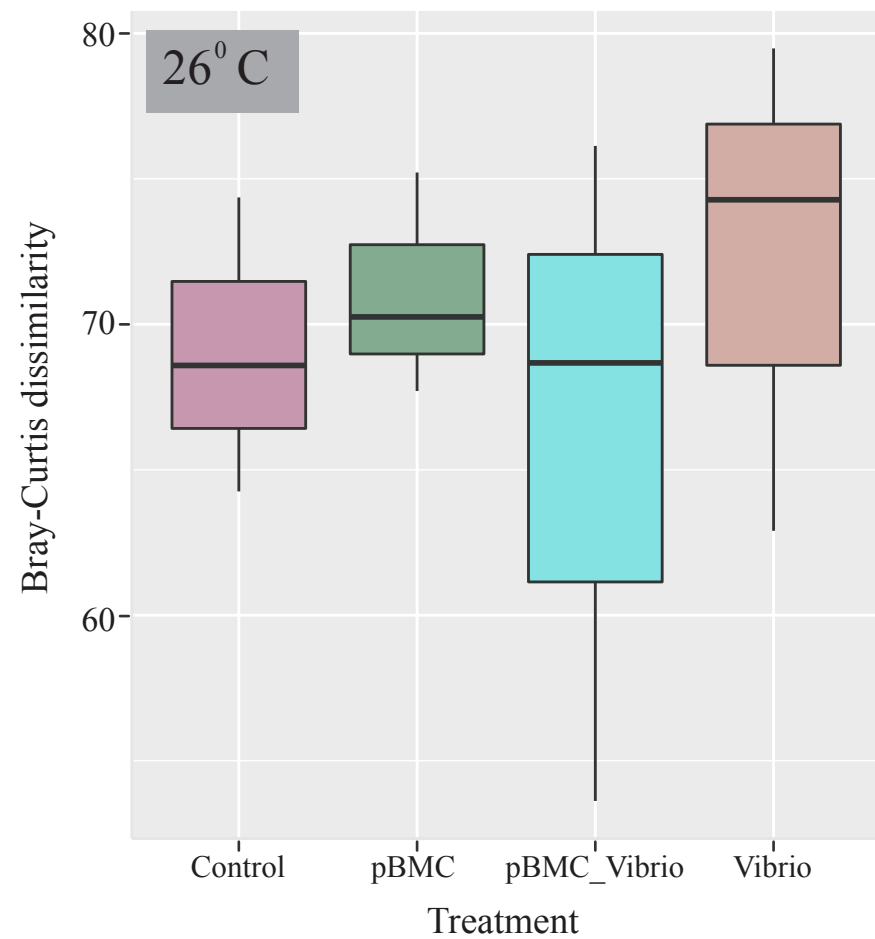

(B)

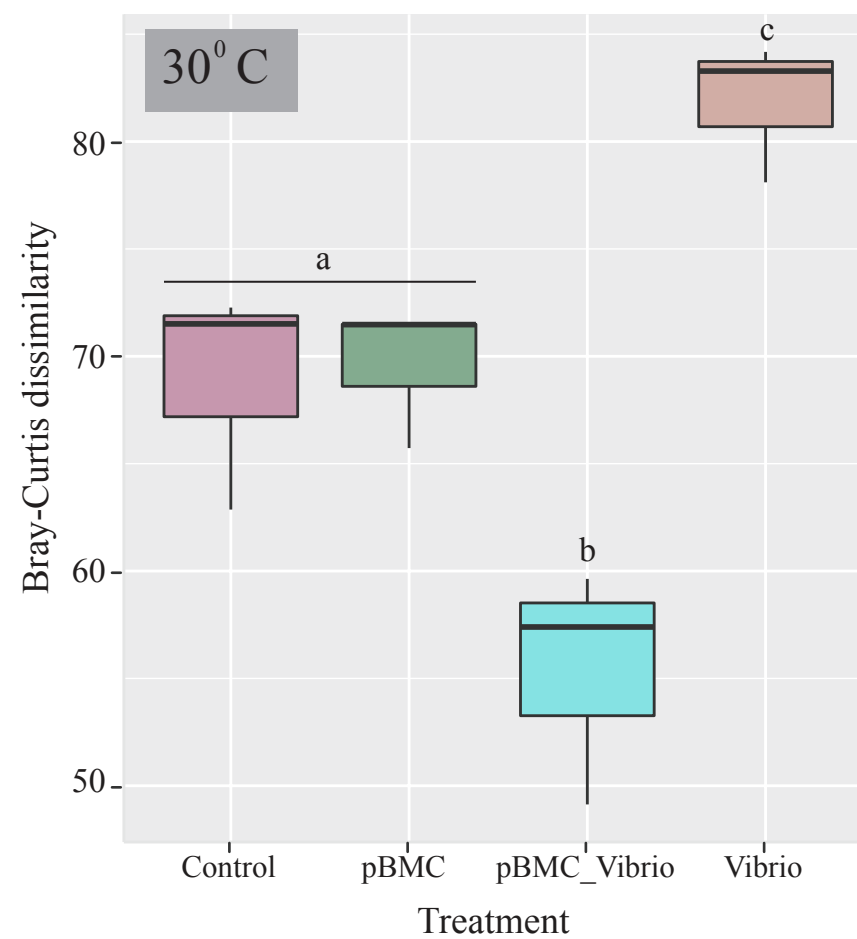

Supplement: Supplementary file 6 — Supplementary Figure 5 [file 41396_2018_323_MOESM6_ESM.pdf]

# Significant ASVs 1, 9 and 26 days at 26° and 30° Celsius

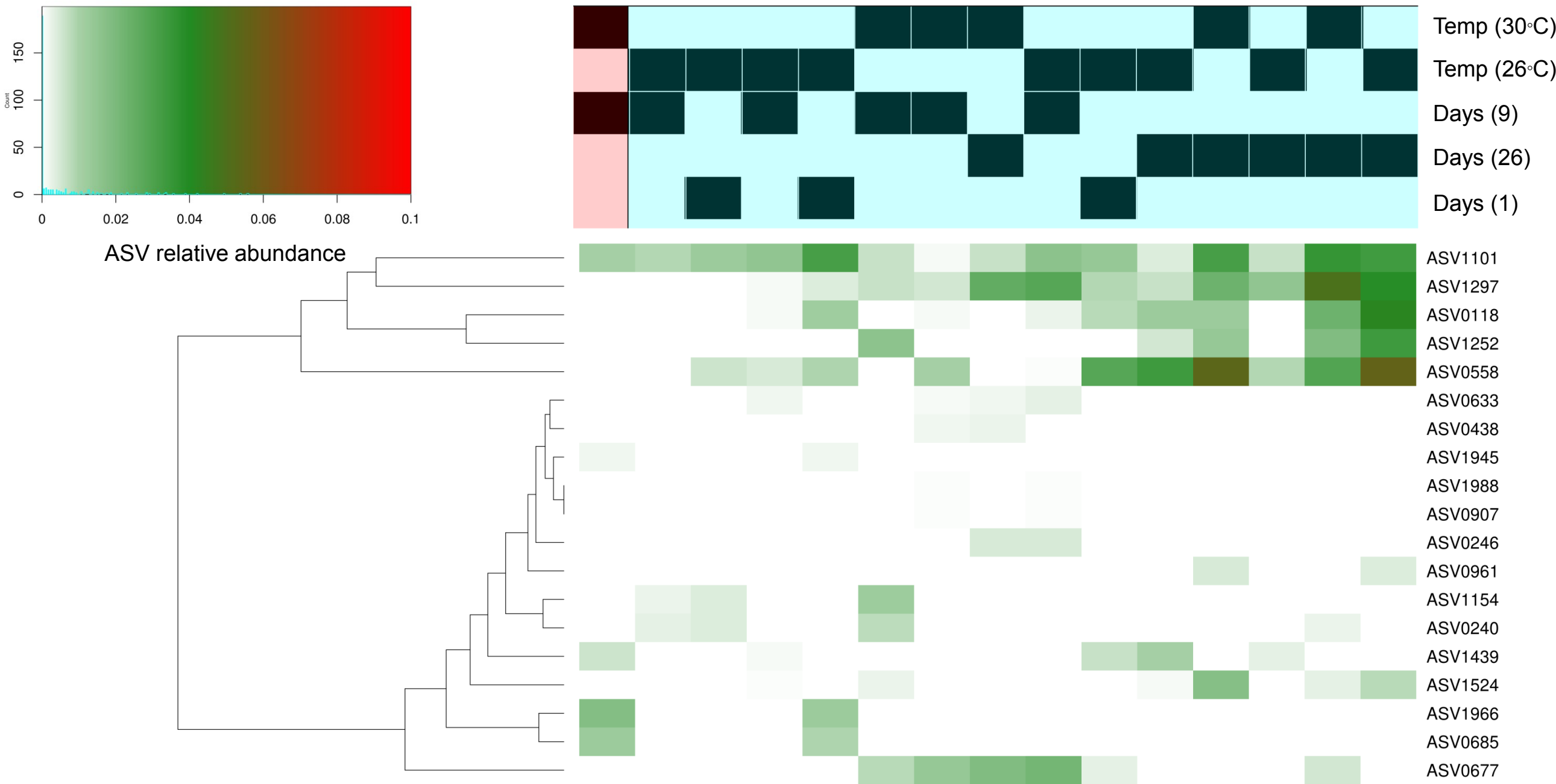

Supplement: Supplementary file 7 — Supplementary Figure 6 [file 41396_2018_323_MOESM7_ESM.pdf]

Significant ASVs 26 days at 26° Celsius

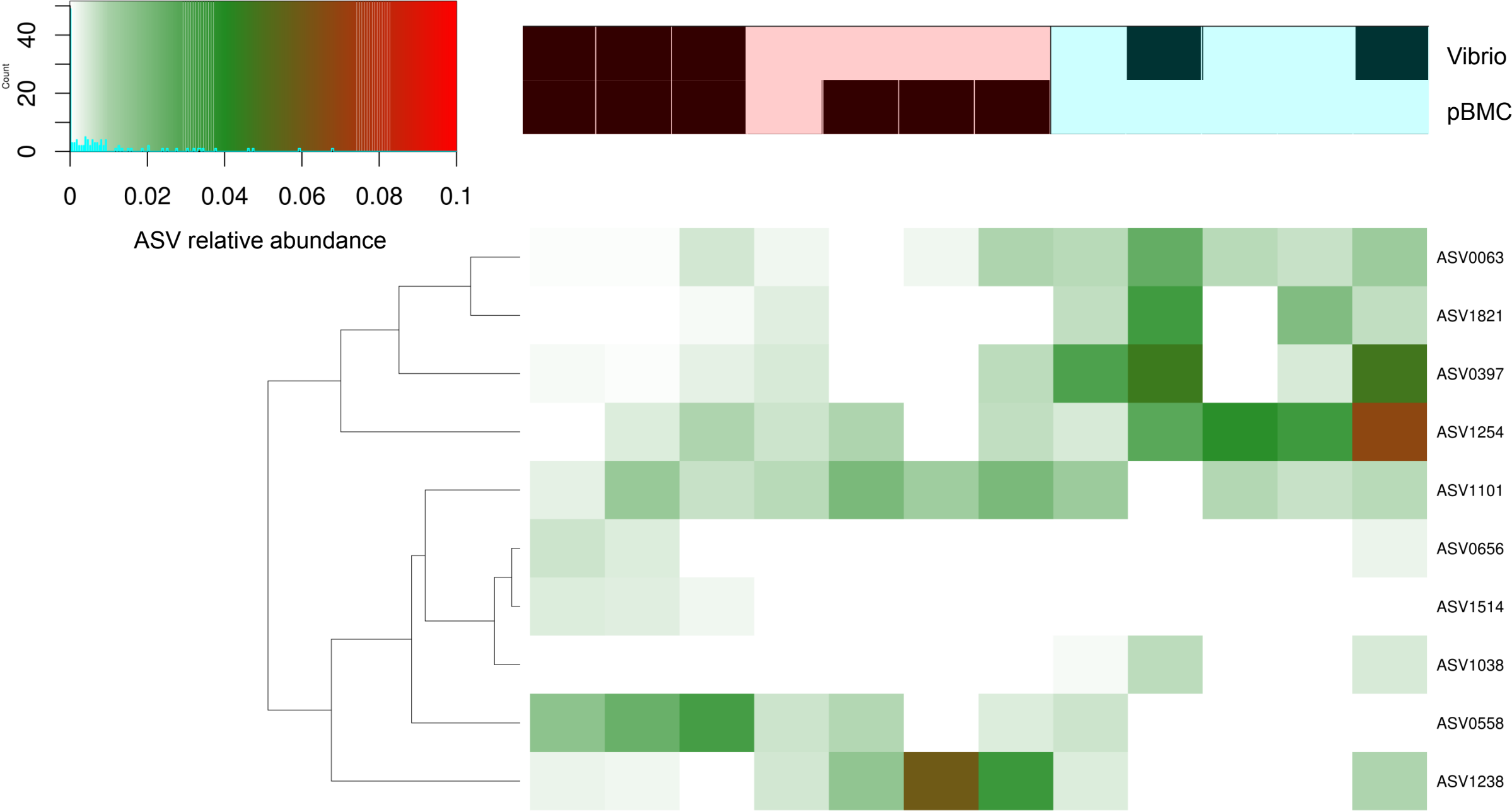

Supplement: Supplementary file 8 — Supplementary Figure 7 [file 41396_2018_323_MOESM8_ESM.pdf]

Significant ASVs 26 days at 30° Celsius

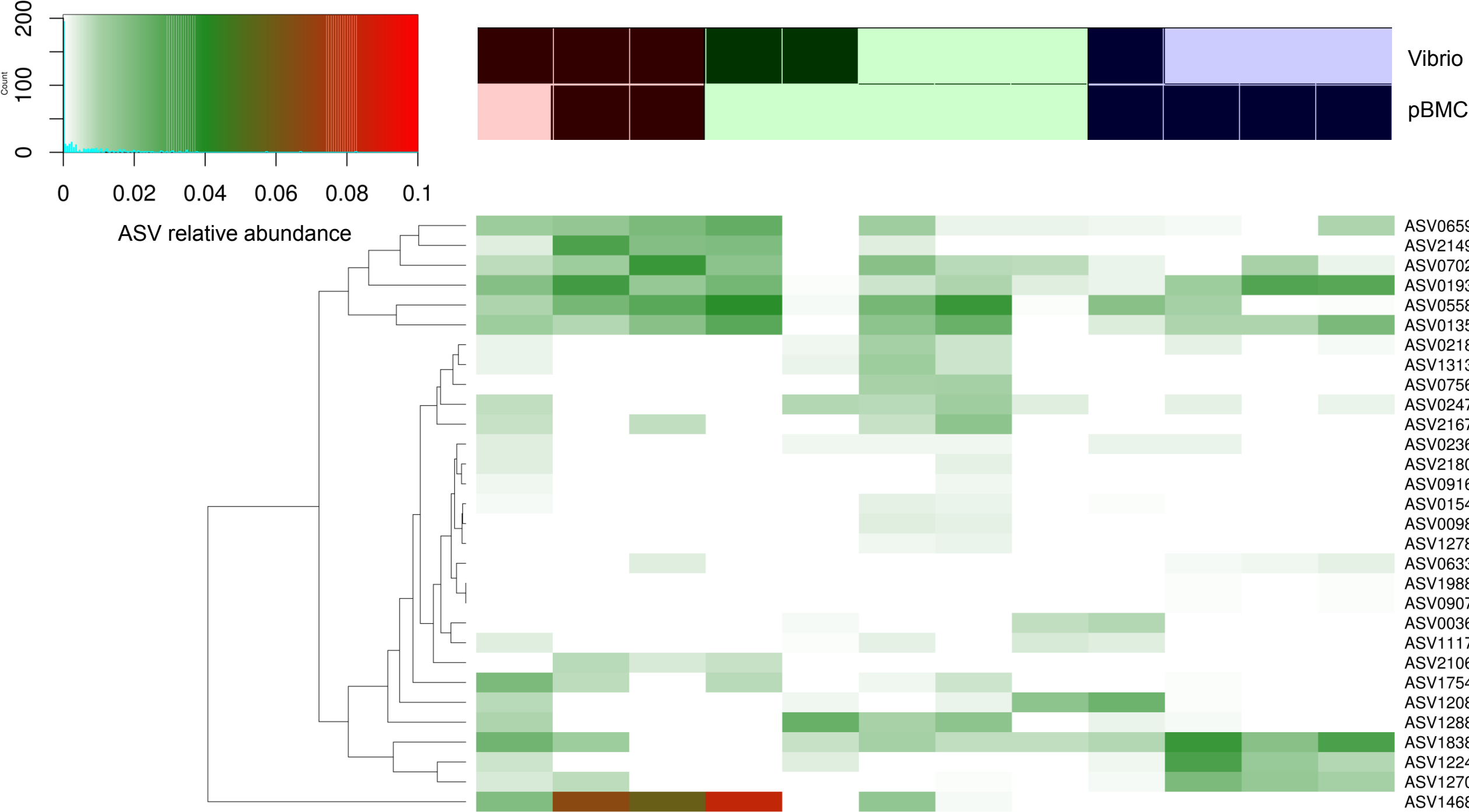

Supplement: Supplementary file 9 — Supplementary Figure 8 [file 41396_2018_323_MOESM9_ESM.pdf]

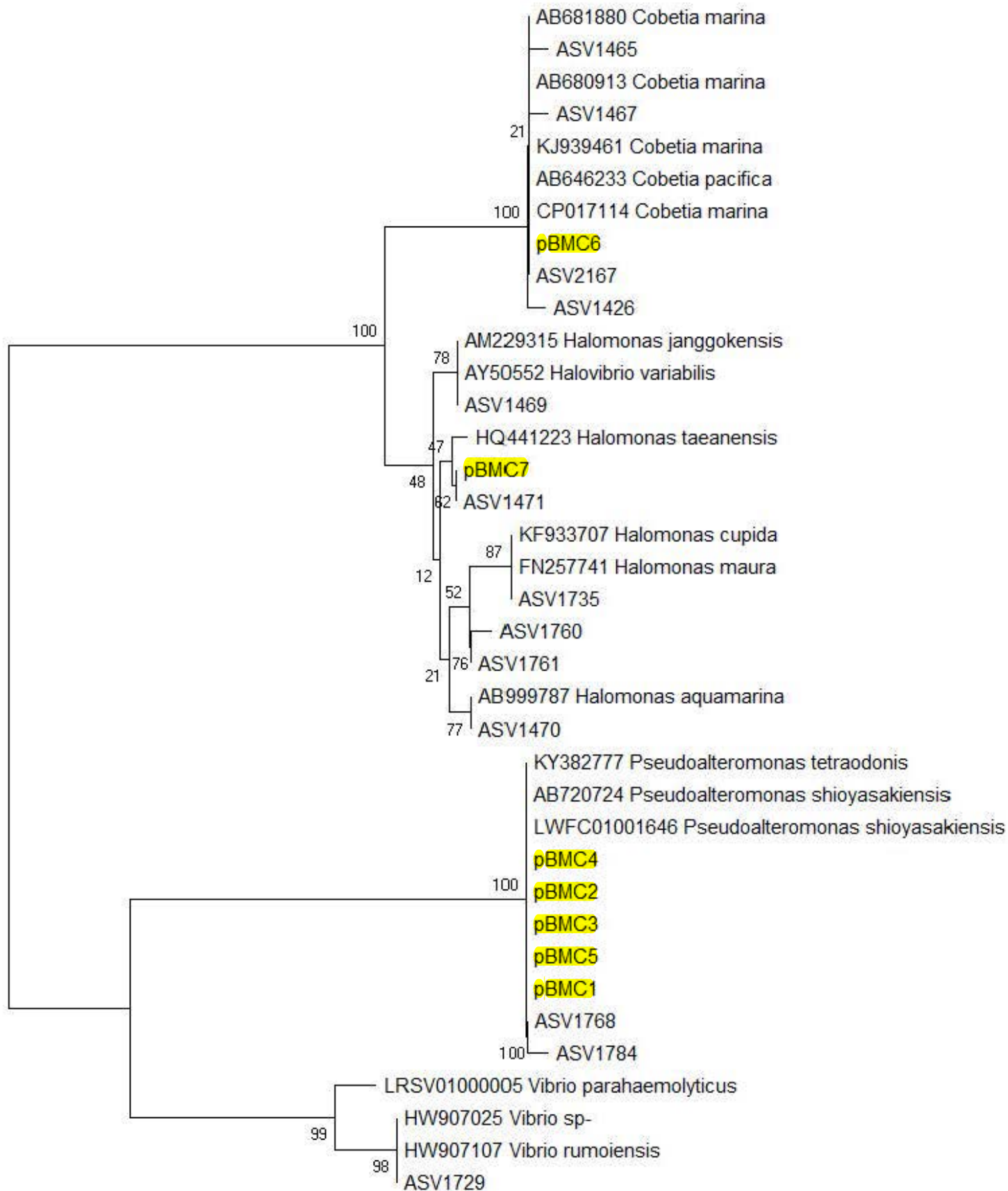

0.020

pBMC 1-5

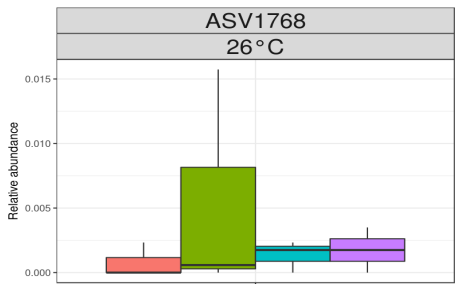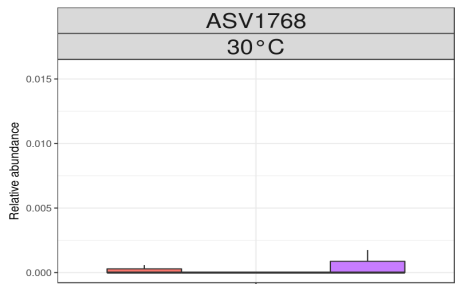

pBMC 6

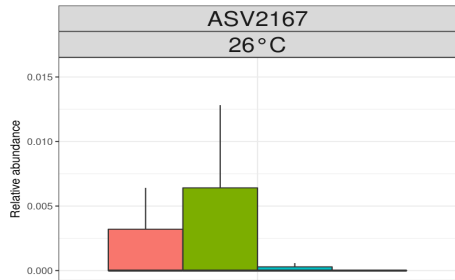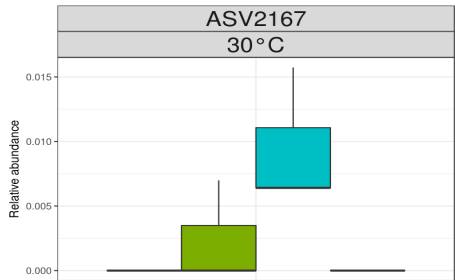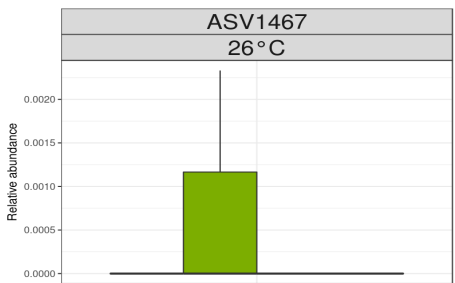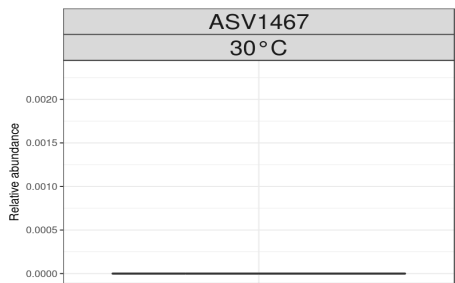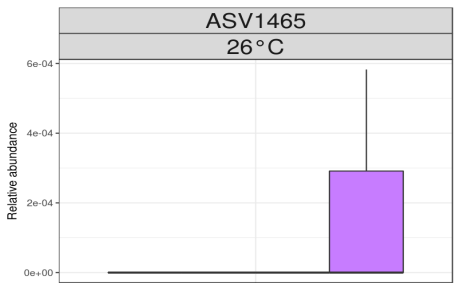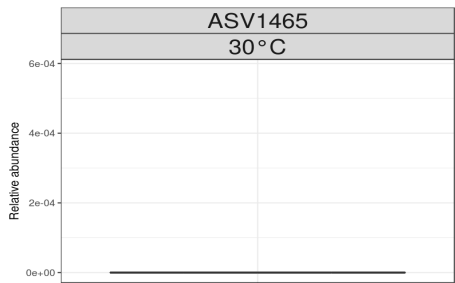

pBMC 7

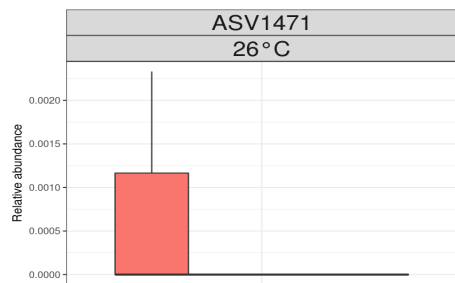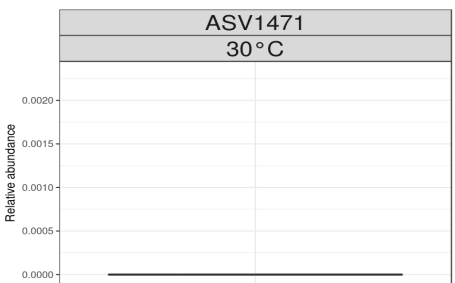

Treatment Control pBMC pBMC\_Vibrio Vibrio

Supplement: Supplementary file 14 — Supplementary Figure 13 [file 41396_2018_323_MOESM14_ESM.pdf]
