# Supplementary material for: Marine probiotics: increasing coral resistance to bleaching through microbiome manipulation
Source: ISME J. 2018 Dec 5;13(4):921–36. doi: 10.1038/s41396-018-0323-6 (PMC6461899; doi:10.1038/s41396-018-0323-6)
Supplement: Supplementary file 10 — Supplementary Figure S9 [file 41396_2018_323_MOESM10_ESM.pdf]

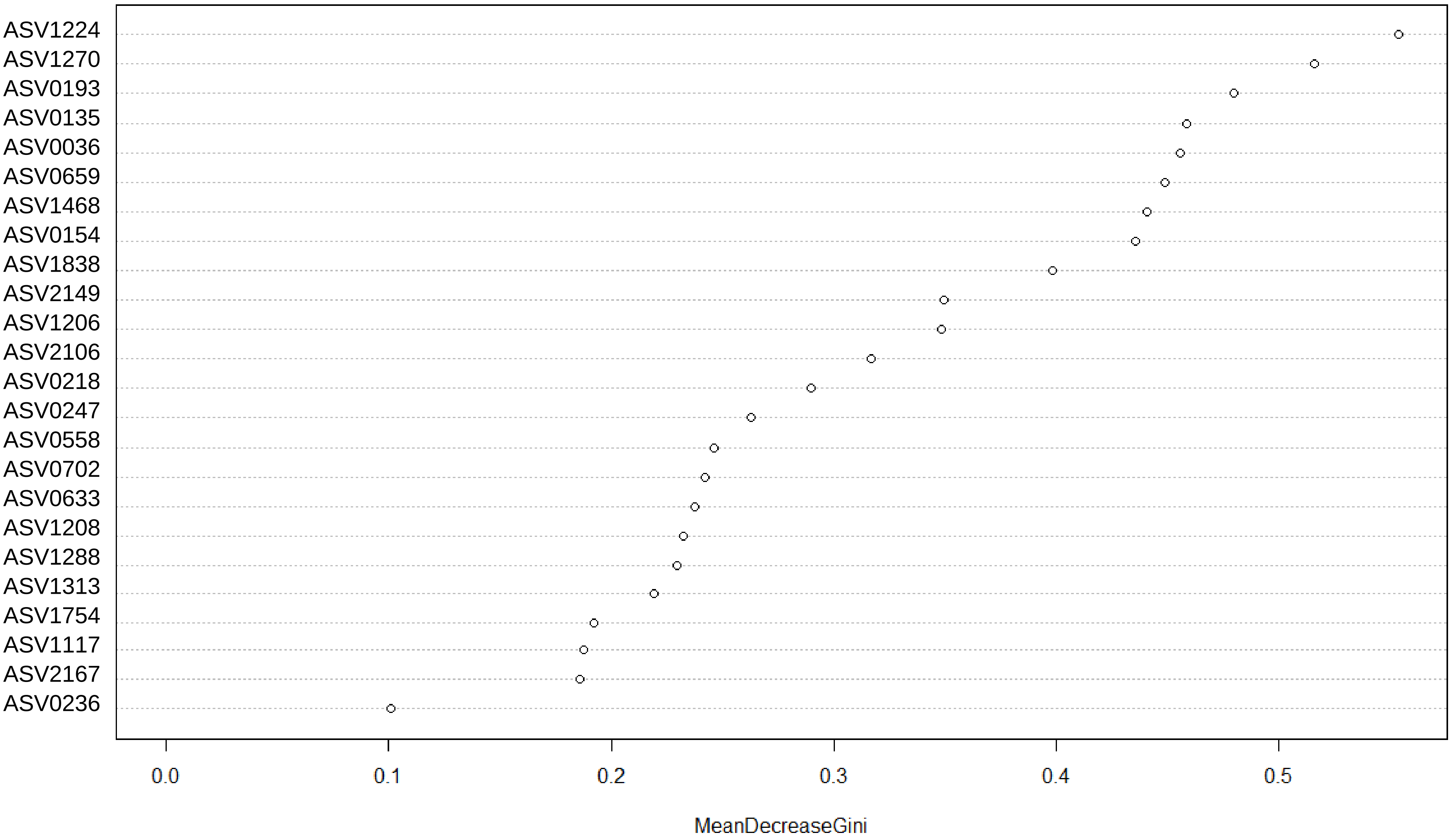

ASV1224
ASV1270
ASV0193
ASV0135
ASV0036
ASV0659
ASV1468
ASV0154
ASV1838
ASV2149
ASV1206
ASV2106
ASV0218
ASV0247
ASV0558
ASV0702
ASV0633
ASV1208
ASV1288
ASV1313
ASV1754
ASV1117
ASV2167
ASV0236
0.0
0.1
0.2
0.3
0.4
0.5
MeanDecreaseGini

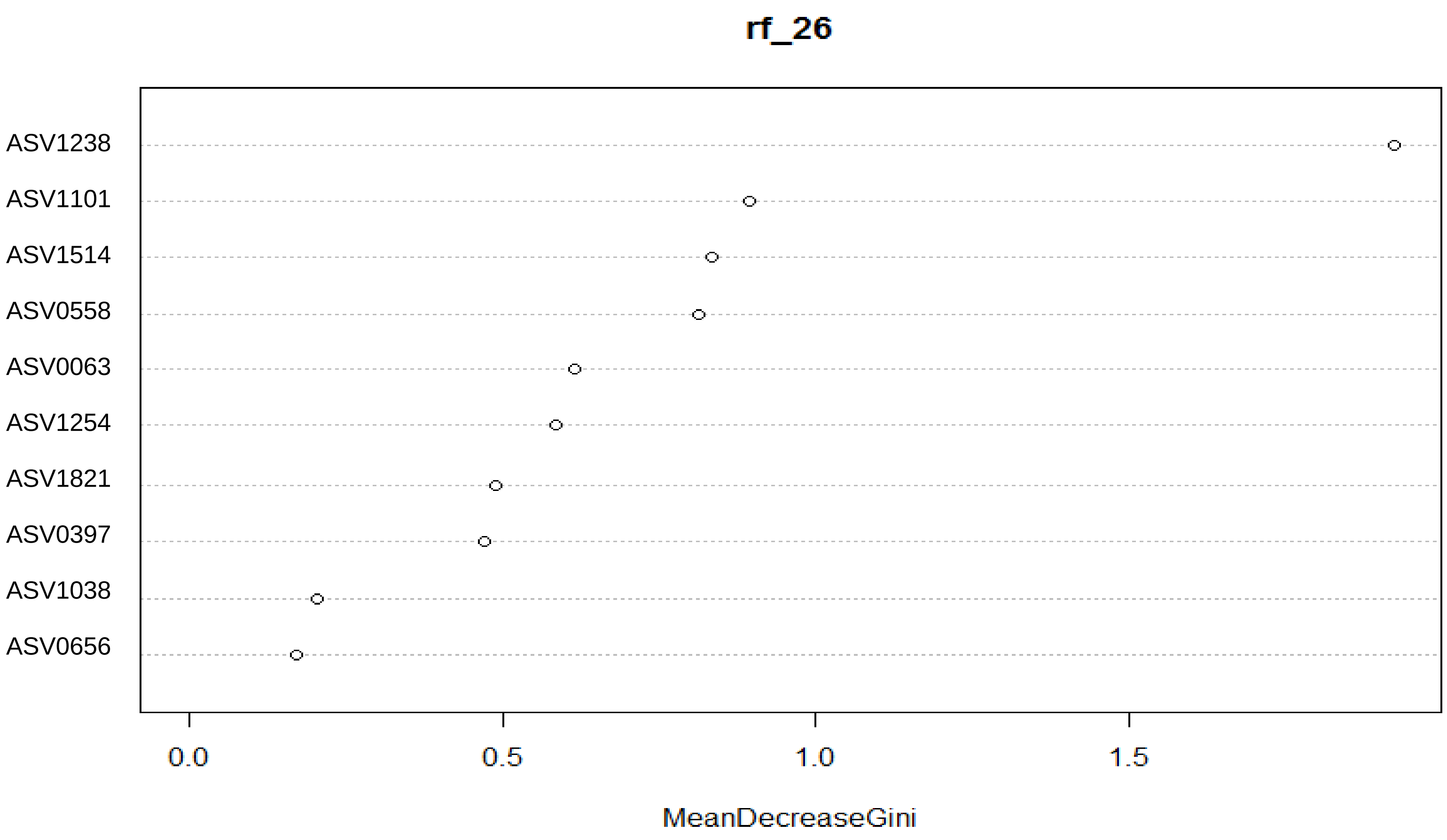
rf_26
ASV1238
ASV1101
ASV1514
ASV0558
ASV0063
ASV1254
ASV1821
ASV0397
ASV1038
ASV0656
0.0
0.5
1.0
1.5
MeanDecreaseGini

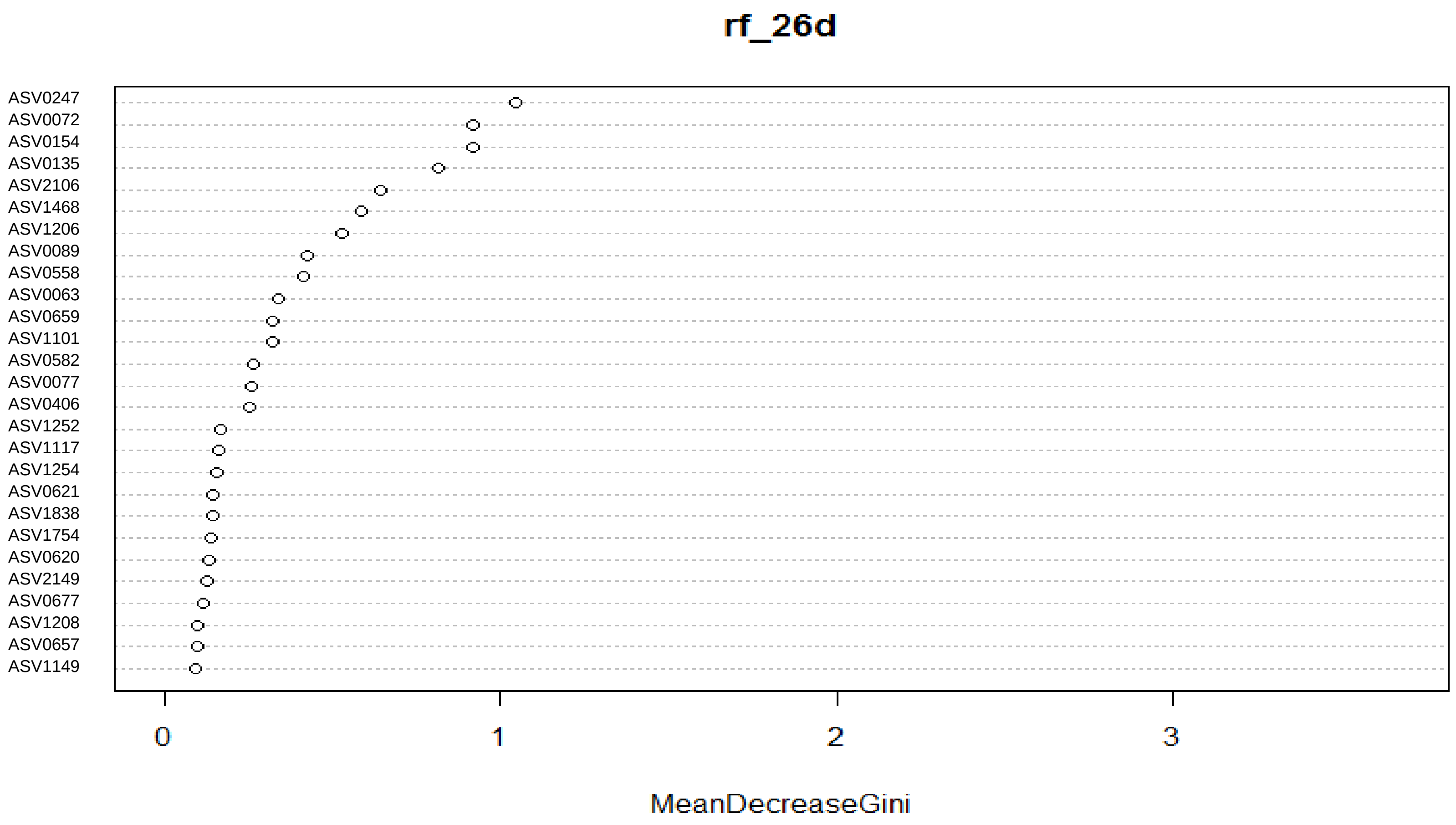
rf_26d
ASV0247
ASV0072
ASV0154
ASV0135
ASV2106
ASV1468
ASV1206
ASV0089
ASV0558
ASV0063
ASV0659
ASV1101
ASV0582
ASV0077
ASV0406
ASV1252
ASV1117
ASV1254
ASV0621
ASV1838
ASV1754
ASV0620
ASV2149
ASV0677
ASV1208
ASV0657
ASV1149
0
1
2
3
MeanDecreaseGini
