# Supplementary material for: Marine probiotics: increasing coral resistance to bleaching through microbiome manipulation
Source: ISME J. 2018 Dec 5;13(4):921–36. doi: 10.1038/s41396-018-0323-6 (PMC6461899; doi:10.1038/s41396-018-0323-6)
Supplement: Supplementary file 12 — Supplementary Figure 11 [file 41396_2018_323_MOESM12_ESM.pdf]

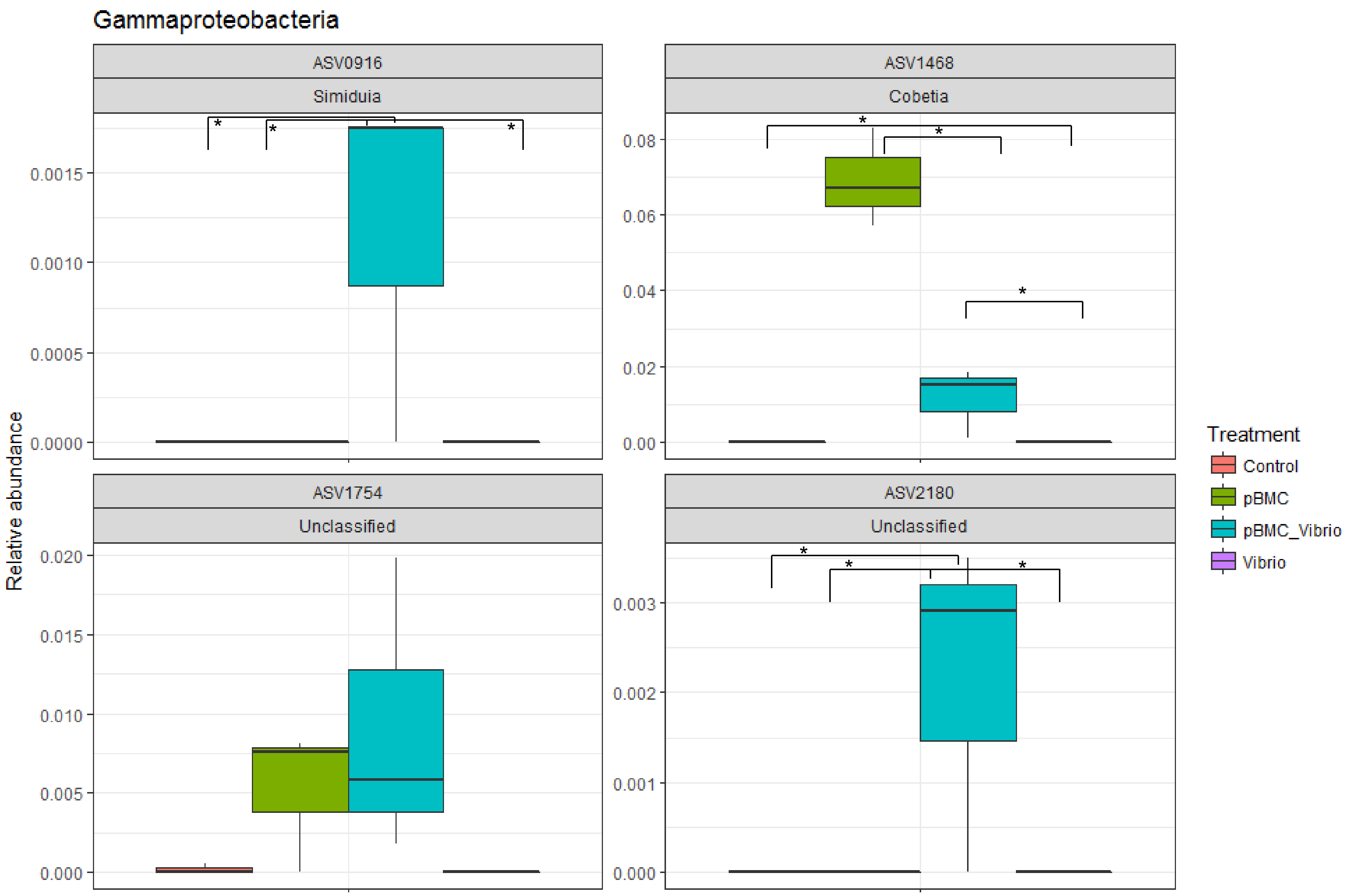

Significant differences of ASV relative abundances using a two way ANOVA with FDR multiple correction testing : * P-value ≤ 0.05, ** P-value ≤ 0.01,*** P-value ≤ 0.001
