# Supplementary material for: Marine probiotics: increasing coral resistance to bleaching through microbiome manipulation
Source: ISME J. 2018 Dec 5;13(4):921–36. doi: 10.1038/s41396-018-0323-6 (PMC6461899; doi:10.1038/s41396-018-0323-6)
Supplement: Supplementary file 15 — Table S1 [file 41396_2018_323_MOESM15_ESM.pdf]

Supplementary Table S1. Selected *Pocillopora damicornis* pBMC strains obtained by pBMC-PCR screening and strain typing by 16S phylogenetic results.

Strain	16S rRNA gene identification	Identity	*nifH*	Catalase	*nirK*	*dmdA*	Antagonistic activity*
**pBMC 1**	*Pseudoalteromonas sp.* (KP016625.1)	99%		+++	X		
**pBMC 2**	*Pseudoalteromonas sp.* (KU955357.1)	100%		+	X		
**pBMC 3**	*Pseudoalteromonas sp.* (KX806641.1)	100%	X	+	X		
**pBMC 4**	*Pseudoalteromonas sp.* (KX806641.1)	100%		+++		X	
**pBMC 5**	*Pseudoalteromonas sp.* (KU955357.1)	99%		+	X		X
**pBMC 6**	*Cobetia marina* (KP236304.1)	99%		++++			
**pBMC 7**	*Halomonas taeanensis* (KJ161492.1)	100%	X	+			

*Antagonist activity was measured agains *V. corallilyticus* DSM19607, see Materials and Methods
