# Supplementary material for: Marine probiotics: increasing coral resistance to bleaching through microbiome manipulation
Source: ISME J. 2018 Dec 5;13(4):921–36. doi: 10.1038/s41396-018-0323-6 (PMC6461899; doi:10.1038/s41396-018-0323-6)
Supplement: Supplementary file 16 — Table S2 [file 41396_2018_323_MOESM16_ESM.pdf]

Supplementary Table S2. Physical-chemical parameters of the water (average). Salinity, pH and dissolved oxygen (OD) were measured every two days (n = 3).

Treatments	Salinity (ppt)	Dissolved Oxygen (mg/L)	pH
**Control 26°C**	34 - 35	6.2 – 6.4	8,05 – 8,1
**pBMC 26°C**	34 - 35	6.2 – 6.4	8,05 – 8,1
**pBMC + VC 26°C**	34 - 35	6.2 – 6.4	8,05 – 8,1
**VC 26°C**	34 - 35	6.2 – 6.4	8,05 – 8,1
**Control 30°C**	34 - 35	5.7 - 6	8,05 – 8,1
**pBMC 30°C**	34 - 35	5.7 - 6	8,05 – 8,1
**pBMC + VC 30°C**	34 - 35	5.7 - 6	8,05 – 8,1
**VC 30°C**	34 - 35	5.7 - 6	8,05 – 8,1
