# Supplementary material for: Marine probiotics: increasing coral resistance to bleaching through microbiome manipulation
Source: ISME J. 2018 Dec 5;13(4):921–36. doi: 10.1038/s41396-018-0323-6 (PMC6461899; doi:10.1038/s41396-018-0323-6)
Supplement: Supplementary file 17 — Table S3 [file 41396_2018_323_MOESM17_ESM.pdf]

Supplementary Table S3. Significant differences in color changes of corals between different treatments after temperature increase from 26° to 30° Celsius determined by Student's t-test (Significant differences: *P<0.05).

Treatment	Control	VC	pBMC	pBMC+VC
**Control**				
**VC**	0.016*			
**pBMC**	0.116	0.013*		
**pBMC+VC**	0.373	0.024*	0.518	

Corals inoculated with: VC (*Vibrio corallilyticus*); pBMC (putatively beneficial microorganisms for corals consortium); pBMC+VC (putatively beneficial microorganisms for corals consortium in conjunction with *Vibrio corallilyticus*)
