# Supplementary material for: Marine probiotics: increasing coral resistance to bleaching through microbiome manipulation
Source: ISME J. 2018 Dec 5;13(4):921–36. doi: 10.1038/s41396-018-0323-6 (PMC6461899; doi:10.1038/s41396-018-0323-6)
Supplement: Supplementary file 18 — Table S4 [file 41396_2018_323_MOESM18_ESM.pdf]

Supplementary Table S4. Variance of rarefied ASV relative abundances of the control samples collected on days 1, 9 and 26 days at 26 and 30° Celsius determined by multivariate permutation analysis[a].

Group interactions	P-value	Residuals
**Temperature**	0.092907	
**Days**	0.000999*	0.58689
**Temperature:Days**	0.287712	

* Statistically significant differences in sample's relative abundance (p-value ≤ 0.05).

[a] Multivariate permutation analysis performed by "adonis" function in "vegan" R package using 1000 permutations.
