# Supplementary material for: Marine probiotics: increasing coral resistance to bleaching through microbiome manipulation
Source: ISME J. 2018 Dec 5;13(4):921–36. doi: 10.1038/s41396-018-0323-6 (PMC6461899; doi:10.1038/s41396-018-0323-6)
Supplement: Supplementary file 19 — Table S5 [file 41396_2018_323_MOESM19_ESM.pdf]

Supplementary Table S5. Pair-wise comparisons of coral reef bacterial community within and across treatments analysed using PERMANOVA (Bray-Curtis distance) with 10^3 permutations. Abbreviations: *P*(perm), *P*-value permutations; *P*(MC) *P*-value Monte-Carlo tests.

**Pair-wise tests**	**t**	***P*(perm)**	***P*(MC)**
**26°C**			
Control : pBMC	0.919	1.000	0.530
Control : Vibrio	0.951	0.800	0.493
Control : pBMC+Vibrio	1.042	0.400	0.394
pBMC : Vibrio	1.085	0.201	0.351
pBMC : pBMC+Vibrio	1.072	0.201	0.369
Vibrio : pBMC+Vibrio	1.138	0.202	0.308
**30°C**			
Control : pBMC	1.302	0.100	0.186
Control : Vibrio	1.185	0.104	0.270
Control : pBMC+Vibrio	1.201	0.103	0.258
pBMC : Vibrio	1.303	0.096	0.185
pBMC : pBMC+Vibrio	1.331	0.105	0.177
Vibrio : pBMC+Vibrio	1.124	0.201	0.319
**Across treatments**			
Control 26°C : Control 30°C	1.084	0.202	0.359
pBMC 26°C : pBMC 30°C	1.190	0.099	0.266
Vibrio 26°C : Vibrio 30°C	1.230	0.102	0.237
pBMC+Vibrio 26°C : pBMC+Vibrio 30°C	1.288	0.103	0.198
