# Supplementary material for: Marine probiotics: increasing coral resistance to bleaching through microbiome manipulation
Source: ISME J. 2018 Dec 5;13(4):921–36. doi: 10.1038/s41396-018-0323-6 (PMC6461899; doi:10.1038/s41396-018-0323-6)
Supplement: Supplementary file 26 — Table S12 [file 41396_2018_323_MOESM26_ESM.docx]

**Supplementary Table S12.** Confusion matrices generated using a random forest algorithm (see materials and methods) for classification of the samples collected on day 26 using the rarefied OTU relative abundances as numeric values and presence and absence of the inoculation of Vibrio and pBMC as factors.

| **Temperature** | **Confusion Matrix** | **Control** | **Vibrio** | **pBMC** | **pBMC + Vibrio** | **Classification error** |
| --- | --- | --- | --- | --- | --- | --- |
| 26^o^C  (41.67%)^a^ | Control | 1 | **2**^b^ |  |  | 0.66 |
|  | Vibrio | **3** |  |  |  | 1.00 |
|  | pBMC |  |  | 3 |  | 0.00 |
|  | pBMC + Vibrio |  |  |  | 3 | 0.00 |
| 30^o^C  (0%) | Control | 3 |  |  |  | 0.00 |
|  | Vibrio |  | 3 |  |  | 0.00 |
|  | pBMC |  |  | 3 |  | 0.00 |
|  | pBMC + Vibrio |  |  |  | 3 | 0.00 |

^a^ Values in between parenthesis represent error rate estimation for the confusion matrices generated for samples at 26^o^C and 30^o^C.

^b^ Values in bold represent the number of misclassified samples.
